# Human limits in machine learning: prediction of potato yield and disease using soil microbiome data

**DOI:** 10.1186/s12859-024-05977-2

**Published:** 2024-11-26

**Authors:** Rosa Aghdam, Xudong Tang, Shan Shan, Richard Lankau, Claudia Solís-Lemus

**Affiliations:** 1 Wisconsin Institute for Discovery, University of Wisconsin-Madison, Madison, WI USA; 2 Department of Plant Pathology, University of Wisconsin-Madison, Madison, WI USA

**Keywords:** Soil microbiome, Phenotype prediction, Microbiome networks analysis, Machine learning, Bayesian neural networks

## Abstract

**Background:**

The preservation of soil health is a critical challenge in the 21st century due to its significant impact on agriculture, human health, and biodiversity. We provide one of the first comprehensive investigations into the predictive potential of machine learning models for understanding the connections between soil and biological phenotypes. We investigate an integrative framework performing accurate machine learning-based prediction of plant performance from biological, chemical, and physical properties of the soil via two models: random forest and Bayesian neural network.

**Results:**

Prediction improves when we add environmental features, such as soil properties and microbial density, along with microbiome data. Different preprocessing strategies show that human decisions significantly impact predictive performance. We show that the naive total sum scaling normalization that is commonly used in microbiome research is one of the optimal strategies to maximize predictive power. Also, we find that accurately defined labels are more important than normalization, taxonomic level, or model characteristics. ML performance is limited when humans can’t classify samples accurately. Lastly, we provide domain scientists via a full model selection decision tree to identify the human choices that optimize model prediction power.

**Conclusions:**

Our study highlights the importance of incorporating diverse environmental features and careful data preprocessing in enhancing the predictive power of machine learning models for soil and biological phenotype connections. This approach can significantly contribute to advancing agricultural practices and soil health management.

**Supplementary Information:**

The online version contains supplementary material available at 10.1186/s12859-024-05977-2.

## Background

Machine learning (ML) has transformed how scientific research is conducted in recent years. Among the many tasks performed by ML models in our daily lives, researchers have relied on ML to assist in clinical diagnoses [1–3], identification of bacterial phenotypes such as antimicrobial resistance [4], and even identification of objects in space [5]. Recently, the vast evidence of the prediction power of ML models on a wide range of applications has launched the adoption of these models in other domains such as sustainable agriculture where soil health – characterized by a wide range of biological, chemical, and physical properties [6, 7]—is explored as an important driver to predict plant phenotypes, such as disease susceptibility or yield.

The question of whether the improvement of soil health could result in superior crop yield and disease resistance remains open, as researchers have not been able to identify a set of indicators to accurately and robustly predict plant outcomes from soil information. Among all the candidate indicators for soil health, soil microbiome is one that continues to be understudied in its predictive potential in the productivity and resilience of agricultural ecosystems [8]. Amplicon sequencing of highly preserved, phylogenetically informative marker genes, such as the 16 S ribosomal RNA for bacteria and the internal transcribed spacer (ITS) for fungi, has enabled extensive studies on the complexity and diversity of soil microbial communities over the last decade. However, little is still understood about how changes in these microbial communities directly impact plant growth and health. Although it is well-recognized that the soil microbiome is closely related to plant health and productivity [9], most current research focuses on how microbial communities change in response to agricultural management.

Fortunately, ML models can bridge this gap by handling complex, high-dimensional data for predictions without requiring prior knowledge of variable interactions. Since we have little or no knowledge of most species among the thousands contained in the soil microbial communities, it would be beneficial to exploit the power of ML models on microbiome data as they are able to explore the unknown interactions between the microbial communities and plant phenotypes.

Analyzing microbiome datasets with ML has three main challenges: (1) The data is compositional, meaning raw counts are normalized by the total number of reads per sample. Therefore, microbial abundances are not independent, and the use of traditional statistical techniques (such as correlation) might lead to increased false discovery rates [10]. (2) Data are highly sparse, which means that datasets include a large number of operational taxonomic units (OTUs) that are present in a small proportion of samples (or in none at all) [11]. (3) Data are high-dimensional, which means that the number of OTUs are larger than the number of samples, especially in more specific taxonomic orders like Order, Family, and Genus.

Beyond microbiome data challenges, prediction accuracy decreases with imbalanced or inaccurate labels. That is, when we consider the task of prediction, each microbial sample should be labeled by a plant outcome value (say, high yield or low yield). It is common that labels in these datasets are imbalanced with one class representing the majority of observed samples which is denoted as an imbalanced binary classification problem. Furthermore, decisions on the class labels are many times not straight-forward. While diseased plant or non-diseased plant tends to be an indisputable classification, determining what constitutes high versus low yield is up for debate and depends not only on the season and other environmental factors, but also on the specific crop variety.

Here, we explore the potential of ML models in the prediction of plant phenotypes of yield and disease from soil data. We utilize a dataset from potato fields in Wisconsin and Minnesota and focus on the performance of the models while facing data challenges related to binarization, imbalance, compositionality, sparsity and high dimensionality. Furthermore, we test the impact of specific data preprocessing steps such as (1) different normalization and zero replacement strategies to overcome the compositionality and sparsity, (2) different feature selection strategies to overcome the high dimensionality, and (3) data augmentation techniques to overcome the imbalance of the data. In addition, we are also interested in answering the question of whether soil microbiome data alone has enough predictive power to predict the disease and yield phenotypes or whether other information on the soil, such as chemical composition, is necessary for accurate prediction. The answer to this question will inform farmers on the best data collection strategies given that soil microbiome data is expensive. That is, if the soil microbiome data does not provide enough predictive power compared to other (cheaper) soil measurements, then the collection of microbiome data for prediction purposes would be futile.

For our investigation, we have chosen two distinct machine learning models. First, we employ random forests (RF), which have consistently demonstrated superior predictive performance across various domains [12]. Further details about this method can be found in section “Random forest model”. Second, we utilize a Bayesian Neural Network (Bayesian NN), known for its inherent protection against overfitting [13] (See section “Bayesian neural network model”). Given their capacity to capture complex interactions among features, neural networks are valuable, especially when dealing with unknown relationships. However, considering the small sample size and large feature space in our study, traditional back-propagation-based neural network models may exhibit a substantial bias or overfitting [14], hence the need to explore the Bayesian version.

We initially explored a variety of machine learning methods to assess precision across different responses. However, given our goal of finding a generalized model that could reliably predict multiple response types, we selected Random Forest (RF). This decision was supported by the model’s consistently comparable results to the H_2_O AutoML package in Python [15], which considers different machine learning algorithms and selects the best-performing ones (See Fig. 3). Additionally, we employed Bayesian Neural Networks (Bayesian NNs) because of their capacity to model parameter uncertainty and their potential to avoid overfitting, particularly useful when working with small datasets. Initially, we worked with continuous yield values, but due to the models’ inability to predict yield reliably (See Fig. 2), we binarized the response to provide more robust and reliable methods.

Among the main findings, we can highlight that microbiome data alone indeed has predictive power for disease outcomes, especially for pitted scab disease, but not to predict yield. We also find that the most powerful prediction is achieved by combination of environmental information and microbiome data. Among the data preprocessing strategies that we explored, we find that normalization and zero replacement strategies have a huge impact on the prediction power of the models, yet there are strong interaction effects with taxonomic levels, and thus, it is impossible to identify one strategy that outperforms the others. In terms of the model, RF outperforms other supervised classification machine learning models, including Bayesian NN which are more computationally expensive and may not be suitable for datasets with a large number of predictors.

We conclude our investigation with a full model selection (FMS) [16, 17] decision tree approach to identify optimal combinations of normalization, zero replacement, feature selection, and model choices that maximize prediction accuracy for microbiome data analysis. Recommendations include using data augmentation and more specific taxonomic levels like Family and Genus for RF, but more general levels like Phylum without augmentation for Bayesian NN, while also identifying specific normalization methods suitable for both models. All the technical terms used in this study are defined in Table S1.

## Methods

**Fig. 1:**
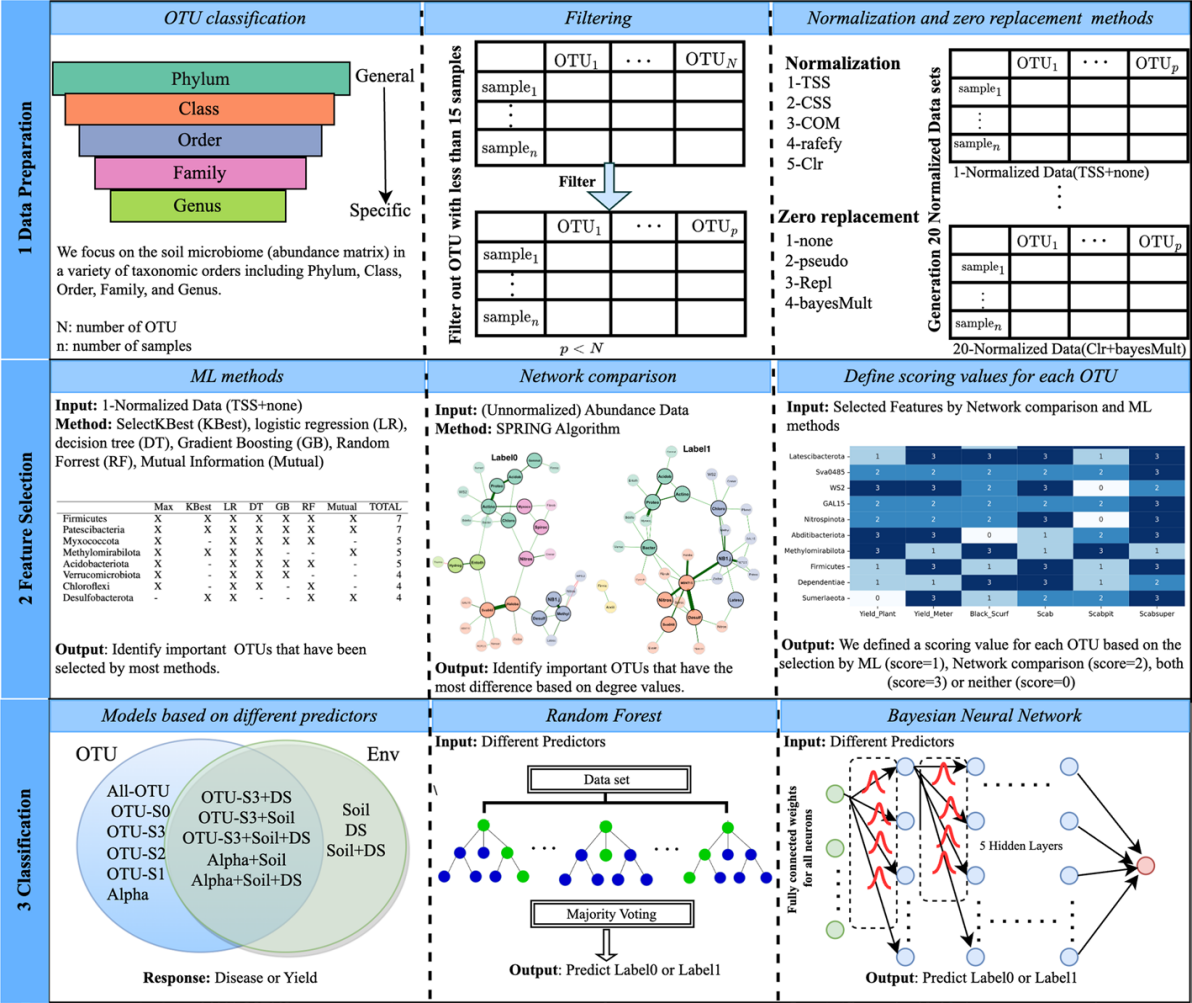
Workflow of the analyses with three main steps: (1) Data Preparation, (2) Feature Selection, and (3) Classification. In (1) Data Preparation, we consider OTUs (number of OTUs = N) at different taxonomic levels and filter by sample size (n). In addition, we perform combination of five normalization methods and four zero replacement methods (for a total of 20 normalized datasets). In (2) Feature selection, we rank OTUs based on i) the number of times they are selected as important features by machine learning (ML) criteria, and ii) the greatest degree of difference on microbial networks reconstructed from samples of each class. We score OTUs based on whether they are selected by ML ($score=1$), by network comparison ($score=2$), both ($score=3$) or neither ($score=0$). In (3) Classification, the Venn diagram depicts the different types of predictors: microbiome (OTUs), environmental (Env), and the combination of both. The acronyms (e.g., All-OTU or OTU-S3+DS) correspond to different choices of predictors that are described in Table 2). random forest and Bayesian neural network classification models are fitted on the different input predictors

Figure 1 shows a graphical representation of our pipeline with three major steps: (1) data preparation, (2) feature selection, and (3) classification based on random forest (RF) and Bayesian NN models with various types of predictors, including microbiome data (OTUs), environmental, and a combination of both. We describe each step in the pipeline in the next subsections.

### Data description and data processing

#### Data description

In this study, we focus on the soil microbiome (matrix of abundances) in a variety of taxonomic orders, including Phylum, Class, Order, Family, and Genus as well as other environmental information from soil samples acquired from potato fields in Wisconsin and Minnesota. The dataset consists of measurements related to soil health, potato yield and soil quality information. The soil health data were collected in the fall of 2019 from pre-planting commercial potato fields and include soil physicochemical properties, soil microbiome composition, soil microbiome diversity, and soil pathogen abundance. Soils were collected from 36 Minnesota fields and 66 Wisconsin fields, with three bulk soil samples randomly selected from each field. The potato yield and quality information at each sampling location was measured at the end of the growing season (September of 2020) including tuber yield and disease severity. Overall, we have 256 samples, 108 of which are taken from fields in Minnesota, and 148 of which are taken from fields in Wisconsin. While this provides a strong foundation for modeling the upper Midwest potato-growing region, we acknowledge that the use of data from a single growing season and geographic region limits the generalizability of our findings. Future data collection from multiple growing seasons and regions is underway to enhance the robustness and applicability of our models. We list all measurements in Table S2 in the Supplementary Material.

**Soil physicochemical properties** Fresh field soils were measured for a variety of physicochemical properties in the Agvise soil testing lab (Benson, Minnesota). Measurements of soil pH, organic matter content, carbon fractions, organic nitrogen, macro and micronutrients are described in [18]. Soil texture was measured by quantifying the relative amount of sand, silt, and clay using a hydrometer. Soil cation/anion exchange capacity was calculated from the nutrient test results mentioned above, reported as milliequivalents per 100 gs of soil.

**Soil microbial community composition and population abundance** Soil microbial community was characterized by high-throughput sequencing of the bacterial 16 S rRNA gene and fungal ITS2 regions. A subsample of 0.25 g of frozen field soils were extracted with the DNeasy PowerSoil Pro DNA isolation kit (Qiagen, CA). Extracted DNA was used in a two-step PCR reaction [19], with the V3-V4 region of bacterial 16 S rRNA and the eukaryotic ITS2 region amplified using the primer set V3F and 806R, and 5.8S and ITS4, respectively [19, 20]. The final PCR product was normalized, pooled and cleaned-up before sequenced on a Illumina MiSeq platform at the University of Minnesota Genomics Center. Sequences were analyzed using Qiime2. Cutadapt was first used to remove the forward and reverse primers of the ITS reads. Trimmed ITS reads and the raw 16 S reads were then truncated, filtered, denoised, pair-end merged, and chimeras removed using the DADA2 pipeline. Taxonomy was assigned to the feature table of amplicon sequence variant (ASV) using Qiime2’s feature-classifier plugin, using the RDP Naïve Bayesian Classifier fit to the SILVA 138 database for 16 S reads and UNITE database for ITS reads. Bacterial and fungal ASV tables were merged at Phylum, Class, Order, Family, and Genus levels using phyloseq in R. Alpha diversity measured as Chao1, Abundance-based coverage estimator, Shannon, Simpson, and Inverse Simpson index were calculated after rarefying the samples to the minimum sample depth. Alpha diversity was calculated for each taxonomic level using the vegan package [21]. The population abundance of bacteria, fungi, *Verticillium dahliae*, and Pathogenic *Streptomyces* were measured with quantitative polymerase chain reaction as described in [18].

**Yield and disease** Potatoes were harvested by hand from a one-meter hill (usually 3-4 plants) at each sampling location at the end of the growing season. One plant was used for tuber disease assessment, and the rest plants were used for yield estimation. Tubers were visually assessed for common scab, silver scurf, and black scurf, and then cut-open to evaluate *Verticillium dahliae* infection (dark vascular ring), and hollow heart. Tuber yield was estimated as the fresh weight of cleaned tubers.

#### Binarization of response variables

We have six phenotypes (response variables) of interest, four of them correspond to diseases and two of them correspond to yield (Table 1).

**Table 1:** Description of responses in this study

Response	N. samples	N. ones	Description
*Yield_Meter*	196	96	Weight of potatoes (grams) per one meter yielded at harvest time
*Yield_Plant*	219	108	Weight of potatoes (grams) per plant yielded at harvest time
*Scab*	231	170	Number of tubers that carry scab (superficial+pitted) disease on the sampled plant
*Scabpit*	231	100	Number of tubers that carry pitted scab disease on the sampled plant
*Scabsuper*	231	145	Number of tubers that carry superficial scab disease on the sampled plant
*Black_Scurf*	231	22	Percentage coverage of black scurf disease on the sampled plant

The first column displays the responses, the second column shows the number of samples, and the third column indicates the count of positive instances (ones) in our binary responses. The last column provides a description of the responses

All six responses in the dataset are continuous, so we need to binarize them to fit the classification models. For the disease-related responses, we simply make the binary label 0 if there is no presence of disease, and 1 if there was detection of disease (that is, if the continuous response is greater than 0.0). Binarizing the yield response variables is harder as there is no universal standard to classify potato yield to be low or high. Furthermore, yield values are highly dependent on the type of potato variety. We assign a label of 0 (low yield) to samples with a yield less than the variety-specific median. Similarly, we assign a label of 1 (high yield) to samples with a yield greater than the variety-specific median. We illustrate this approach in Fig. S1 in the Supplementary Material.

After binarization, we note that pitted scab disease (denoted *Scabpit* in the figures), superficial scab disease (denoted *Scabsuper* in the figures), and both yield responses are balanced, whereas other responses are highly imbalanced: scab disease (denoted *Scab* in the figures) has 80% of samples labeled as 1, and black scurf disease (denoted *Black_Scurf* in the figures) has only six samples labeled as 1. We use these imbalanced cases to assess the performance of the methods under imbalance settings and data augmentation strategies.

#### Data filtering, normalization, and zero replacement

The input data is a matrix with non-negative read counts that were generated by a sequencing procedure. Let $w^{(k)}= [w_1^{(k)},...,w_p^{(k)}]$ be the total read counts of sample *k* containing *p* OTUs, where $w^{(k)}$ is a composition that adds up to a fixed value of $m^{(k)}=\sum _{i=1}^p w_i^{(k)}$. This value $m^{(k)}$ is the sequencing depth, which varies across samples and is predetermined by technical factors resulting in highly sparse data. It is reasonable to filter out a certain set of OTUs as the first data preparation step. For filtering, we only include OTUs that appear in at least 15 samples. Table S3 displays the number of features (OTUs) before and after filtering for different taxonomic levels.

As mentioned, the input data is compositional and highly sparse. It is known that ML methods do not perform well with unnormalized data [22] and with sparse data [23]. Therefore, we explore the effect of four zero replacement strategies (to overcome sparsity) and five normalization strategies (to overcome compositionality). All strategies are implemented in the NetCoMi R package [24].

In particular, we consider the four zero replacement strategies: (1) the original dataset which included zeros (denoted *none* in the figures), (2) pseudo-zero replacement which replaces zero counts by a predefined pseudo count (denoted *pseudo* in the figures), (3) multiplicative zero replacement which imputes left-censored compositional values by a given fraction and applies a multiplicative adjustment to preserve the multivariate compositional properties of the samples (denoted *multRepl* in the figures) [25], and (4) Bayesian-multiplicative treatment which imputes zero counts by posterior estimates of the multinomial probabilities generating the counts, assuming a Dirichlet prior distribution (denoted *bayesMult* in the figures) [26].

Next, we use five normalization methods: (1) Total sum scaling which simply converts counts to appropriately scaled ratios (denoted *TSS* in the figures) [27], (2) Cumulative sum scaling which rescales the samples based on a subset (quartile) of lower abundant taxa, thereby excluding the impact of highly abundant taxa (denoted *CSS* in the figures) [27], (3) Common sum scaling in which counts are scaled to the minimum depth of each sample (denoted *COM* in the figures) [22], (4) Rarefying which random samples without replacement after a minimum count threshold has been applied (denoted *rarefy* in the figures) [28], and (5) Centered Log-ratio which transforms the data using the geometric mean as the reference (denoted *clr* in the figures) [29].

With four zero replacement methods and five normalization methods, we create 20 datasets by the combination of zero replacement and normalization techniques. Our goal is to study the effect of the zero replacement and normalization choice in the performance of the deep learning methods. Namely, we have the following 20 combinations, $\text {NM}_1$: TSS+none, $\text {NM}_2$: TSS+pseudo, $\text {NM}_3$: TSS+multRepl, $\text {NM}_4$: TSS+bayesMult, $\text {NM}_5$: CSS+none, $\text {NM}_6$: CSS+pseudo, $\text {NM}_7$: CSS+multRepl, $\text {NM}_8$: CSS+bayesMult, $\text {NM}_9$: COM+none, $\text {NM}_{10}$: COM+pseudo, $\text {NM}_{11}$: COM+multRepl, $\text {NM}_{12}$: COM+bayesMult, $\text {NM}_{13}$: rarefy+none, $\text {NM}_{14}$: rarefy+pseudo, $\text {NM}_{15}$: rarefy+multRepl, $\text {NM}_{16}$: rarefy+bayesMult, $\text {NM}_{17}$: clr+none, $\text {NM}_{18}$: clr+pseudo,, $\text {NM}_{19}$: clr+multRepl, and $\text {NM}_{20}$: clr+bayesMult. For convenience, we use the notation $\text {NM}_i$ (Normalization Method) for $i=1,\dots ,20$ in the Full Model Selection section (See section “Identifying general practices to predict potato disease from microbiome data using full model selection models”).

For the environmental predictors of soil chemistry and microbial population density in the soil, we apply six scaling methods: (1) standardize features by subtracting the mean and scaling to unit variance [30]; (2) scale each feature to a [0, 1] range; (3) scale each feature by its maximum absolute value; (4) scale features by subtracting the median and scaling to the interquartile range [31]; (5) transform the features to follow a normal distribution [32]; (6) normalize samples individually to the unit norm. After normalization, the datasets are split into training, validation, and testing sets with 10-fold cross-validation. We used 80% of samples for training and validation, and 20% for testing.

#### Data augmentation

There are three main goals that we wish to achieve with data augmentation: (1) improve the model’s prediction performance with more artificial samples; (2) balance the number of labels with artificial samples, and (3) make the model more robust and avoid overfitting with unseen (artificial) data. We note that augmenting the whole dataset and then splitting it into training and testing sets would result in *data leakage*. For example, when the original sample is in the testing set and the augmented sample from this sample is in the training set, the model is essentially training and testing on the same sample since the normalized values of OTUs are very close. Thus, we split the data into training and testing sets first and only augment the training set. This strategy also allows us to have a fair performance comparison for augmented and non-augmented sets with the same testing data.

Regarding the data augmentation procedure, instead of simply adding a randomly generated noise to the original sample, we subset the data by variety and label, compute the mean (and standard deviation) abundance value for this subset, and create a new sample that includes the original data plus a Gaussian error with mean $\mu /100$ and standard deviation $\sigma /100$ where $\mu , \sigma$ are the subset-specific mean and standard deviation, respectively. This approach is illustrated in Fig. S2 in the Supplementary Material. By the end of this procedure we would have a balanced augmented training set with 400 samples per label for each of the five taxonomic levels (Phylum, Class, Order, Family, and Genus), the number of samples is shown in Table 1.

### Feature selection

Feature selection involves the identification of important features (or covariates) that have high predictive power. Given the high-dimensionality of the data (e.g. 256 original samples for 485 OTUs at the Genus level), feature selection is necessary, especially for Bayesian NN models that are computationally expensive.

We pursue two approaches for feature selection: (1) using ML models to assess variable importance, and (2) using network analyses. To focus exclusively on the effect of feature selection, we only consider one type of normalization and zero replacement strategy in this investigation, namely, total sum scaling normalization without zero replacement ($\text {NM}_1$: TSS+none).

#### Using ML models for feature selection

To identify important OTUs, we use six ML strategies implemented in scikit-learn [32]: (1) “SelectKBest” method selects features based on the *k* highest analysis of variance F-value scores, (2) select the top *k* features based on the mutual information statistic, (3) recursive feature elimination (RFE) with logistic regression, (4) RFE with decision tree, (5) RFE with gradient boosting, and (6) RFE with RF. In addition to the six ML strategies, we consider a 7th strategy which consists in including OTUs in the model if their maximum value is within the top 30%.

After running all seven strategies, we assign a value (“TOTAL”) to each OTU based on the number of times the OTU is selected as an important feature under the seven criteria. That is, an OTU that is selected as important by all seven strategies will have a value of 7. The OTUs are sorted based on “TOTAL” column and the top 30% of them are selected as important features. Thus, 30, 36, 75, 85, and 162 OTUs are selected for Phylum, Class, Order, Family, and Genus levels, respectively. For example, Table S5 in the Supplementary Material shows the top 30 OTUs at the Phylum level and by which strategies they are identified as important features to predict the pitted scab response.

#### Using network comparison for feature selection

Next, we identify important OTUs by comparing their interactions in microbial networks when the network is constructed with samples from one class (say, low yield) versus with samples from the other class (say, high yield) [10]. Indeed, evidence shows that microbial interactions can help differentiate between crop disease states [24]. We identify the OTUs that interact differently on the samples corresponding to one class versus another class as relevant OTUs that contain information about the outcome. We use the SPRING method (semi-parametric rank-based approach for inference in the graphical model) [33] in the NetComi package to construct two microbial networks: one corresponding to label 0 and one corresponding to label 1. To build these networks, the estimated partial correlations are transformed into dissimilarities via the signed distance metric, and the corresponding similarities are then used as edge weights. We then compare these networks in order to identify OTUs that have the greatest difference based on degree values. Figure S4 shows an example of two microbial networks with different graphical structures, and Table S6 in the Supplementary Material lists OTUs ranked by the difference in degree in the two microbial networks for diseased and non-diseased classes of pitted scab response. The OTUs are sorted based on the values of the degree difference column and similar to ML strategy top 30% of them are selected as important features.

#### Combination of ML and network strategies in feature selection

We define a scoring value for each OTU based on whether they are identified as important by ML strategy ($score=1$), by the network comparison ($score=2$), or both ($score=3$). If the OTU is not identified as important by any strategy, it is denoted $score=0$. Figure S5 displays the scoring values of all OTUs at the Phylum level for all responses.

### Model descriptions: random forest and Bayesian neural network

We apply two types of classification models to predict potato disease and yield: RF and Bayesian NN. We consider separately three types of predictors: OTU abundances (5 taxonomic levels and 20 normalization/zero replacement strategies described in section “Data filtering, normalization, and zero replacement”), environmental predictors such as soil characteristics and microbial population density, and a combination of both types. Table 2 lists all the models we consider in this study.

**Table 2:** Models under study for three types of predictors (first column)

Type of predictors	Name of the model	Predictors included in the model
OTU	ALL-OTU	OTU abundances
	OTU-S0	OTUs with a score of zero (not selected by ML or network comparison feature selection strategies)
	OTU-S1	OTUs selected by the ML feature selection strategy (score of one)
	OTU-S2	OTUs selected by the network comparison feature selection strategy (score of two)
	OTU-S3	OTUs selected by the ML and network comparison feature selection strategies (score of three)
	Alpha	Alpha diversity
Environmental	Soil	Soil chemistry
	DS	Microbial population density in soil
	Soil+DS	Combination of soil chemistry and microbial population density in soil
Combination	Alpha+Soil	Combination of alpha diversity and soil chemistry
	Alpha+Soil+DS	Combination of alpha diversity, soil chemistry, and microbial population density in soil
	OTU-S3+Soil	Combination of OTUs with score of three and soil chemistry
	OTU-S3+DS	Combination of OTUs with score of three and microbial population density in soil
	OTU-S3+Soil+DS	Combination of OTUs with score of three, soil chemistry, and microbial population density in soil

*OTUs* environmental, and both. The name of each model (second column) is used in figures and tables in the text

Table S4 shows the number of predictors included in each model as well as the data choices related to taxonomic level or normalization and zero replacement strategies. For example, using all OTUs (first row), we have five taxonomic levels and 20 normalization+zero replacement strategies each, so in total, we have 100 (20 normalization strategies times 5 taxonomic levels) different OTU datasets. For each of these 100 datasets, the number of predictors (i.e., OTUs) would depend on the taxonomic level being analyzed. For example, at the Phylum level, there might be 42 predictors, while at the Genus level, there could be up to 485 predictors.

#### Random forest model

The RF classifier is a powerful ML technique that has gained significant popularity in the last two decades because of its accuracy and speed. RF randomly creates an ensemble of decision trees. Each tree picks a random set of samples (bagging) from the data and models the samples independently from other trees. Instead of relying on a single learning model, RF builds a collection of decision models, and the final decision is based on the output of all the trees in the model. The bagging approach promotes the generation of uncorrelated trees which reduces the risk of overfitting. Each decision tree is generated individually without any pruning and each node is split using a user-defined number of features. By expanding the forest to a user-specified size, the technique generates trees with a high variance and low bias. The final classification choice is determined by summing the class-assignment probability obtained by each tree. A new unlabeled data in testing set input is thus compared to all decision trees formed in the ensemble, with each tree voting for class membership and the membership category with the most votes will be picked.

The RF has several hyperparameters to be determined by the user, such as the number of decision trees to be generated, the number of variables to be selected and tested for the best split when growing the trees, the maximum depth of the tree, the minimum number of samples required to split an internal node, among others. Generally, a grid search is combined with K-fold cross validation to select the best hyperparameters [34]. GridsearchCV is a well-known search method which is available in scikit-learn [32] and it evaluates all possible parameter combinations to determine optimal values.

Here, we set different values for parameters (see Table S7 in the Supplementary Material) and tune them using GridsearchCV to find the optimal values for the RF classifier. GridSearchCV uses a “score” method for evaluating the performance of the cross-validated model on the test set.

For evaluating our results, we employ the weighted F1 score due to the presence of unbalanced data. The weighted F1 score provides a comprehensive evaluation metric that considers both precision and recall across multiple classes, taking into account the class imbalance. It is calculated using this formula: $\text {weighted F1 scores} = \frac{\sum _{i=1}^{N} w_i \cdot \text {F1 score}_i}{\sum _{i=1}^{N} w_i}$

where $N$ is the number of classes, here is 2 and $w_i$ is the weight assigned to class $i$ which is determined based on the size of each class, and $\text {F1 score}_i$ is the F1 score for class $i$. A weighted F1 score of 1 indicates the best possible result, while a score of 0 indicates the worst possible result.

Finally, when the parameters for the RF model are tuned, we use 20% of samples to report the performance of the final model. We use the weighted average F1 score to evaluate the performance of the models, which is computed by averaging all the per-class F1 scores while accounting for the number of samples in each class.

#### Bayesian neural network model

The size of the samples used in our study is too small for traditional Deep Learning approach. Bayesian NN are suitable for small sample sizes as they provide natural protection against overfitting by considering distributions for the model parameters. This is due to the fact that distributions are considered for the parameters in the model which allow us to marginalize them so that the prediction is based on data points alone [13]. In the first paragraph of section “Details of bayesian neural network model” of the Supplementary Material, we provide the mathematical details of Bayesian NN models.

We structure our Bayesian NN models based on the datasets and we set the prior distributions and hyperparameters following the scheme described in [35]. The detailed mathematical representation of the parameters and structure information of our model could be found in the second paragraph of section “Details of bayesian neural network model” of the Supplementary Material.

The training and the approximation of the posterior distribution are done via a Hamiltonian (Hybrid) Monte Carlo (HMC) implemented in [35]. The choice of leap frog lengths and step sizes could be found in the last paragraph of section “Details of bayesian neural network model” of the Supplementary Material.

Given that HMC does not scale well for high dimensional parameter spaces and large datasets [36], we did not fit the Bayesian NN on all the models described in Table 2. In particular, we do not fit a Bayesian NN model on the Genus level for the ALL-OTUs as this model would involve over 9 million weights in the network with 485 input neurons.

### Full model selection

FMS [16, 17, 37] involves the process of listing all data preprocessing steps, model options and selection of predictors, and using a decision tree model to identify the choices that yield the highest measure of performance. Here, we fit a FMS strategy with the following options: 1) type of normalization, 2) type of zero replacement, 3) taxonomic level, and 4) data augmentation. We combine the type of normalization and type of zero replacement strategy into one variable (denoted $\text {NM}_i$ for $i=1,\dots ,20$). We focus on the weighted F1 score as measure of performance, and we include all OTU predictors (that is, we do not consider feature selection as one of the options to compare). We build the regression decision tree by using the DecisionTreeRegressor which is available in scikit-learn [32]. We use the default parameters in the DecisionTreeRegressor such as “squared error” as the criterion to measure the quality of a split, a minimum number of 2 for the samples required to split an internal node, and a minimum number of 1 sample required to be at a leaf node. In order to create an informative decision tree that can be interpreted, we use a maximum depth of 4.

## Results

### Performance evaluation of predictive models for yield responses

**Fig. 2:**
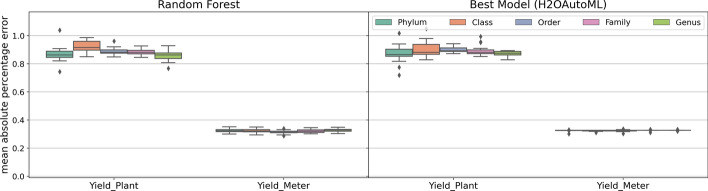
Boxplots with Mean Absolute Percentage Error (MAPE) values: random forest and Best Model resulted from the H_2_OAutoML python package. The x-axis represents the yield outcomes. The range of each box plot depicts the MAPE values for 20 normalized datasets at each taxonomic level

We implemented the H_2_O AutoML package in Python [15], an open-source package designed for automated ML, which trains multiple models such as RF, Gradient Boosting Machines, and Deep Learning models. H_2_O AutoML automates model selection and hyperparameter tuning, providing a comprehensive comparison of different ML methods. The best-performing model is selected based on Root Mean Square Error (RMSE), a standard metric for regression tasks. This process allows for a more robust evaluation of model performance in predicting continuous yield, avoiding the biases introduced by arbitrary binarization. However, to maintain consistency with the rest of the study, we also report results from RF models. We report Mean Absolute Percentage Error (MAPE) as the evaluation metric. MAPE calculates the average absolute difference between predicted and actual values as a percentage of the actual values. The formula for MAPE is defined as:

$$\begin{aligned} \text {MAPE} = \frac{1}{n} \sum _{i=1}^{n} \left| \frac{A_i - F_i}{A_i} \right| \times 100 \end{aligned}$$

where $A_i$ is the actual value, $F_i$ is the predicted value, and n is the number of observations. MAPE, evaluated on a scale from 0 to 1, is commonly used for regression tasks because it is particularly valuable as it is scale-independent, making it easy to compare performance across different models and datasets [38]. A lower MAPE indicates better model accuracy, with 0% representing perfect prediction. A MAPE of, for example, 10% means that, on average, the model’s predictions are 10% off from the actual values. The results of MAPE values are visualized using box plots, which clearly represent the variability and performance of the models across different normalization methods and taxonomic levels in Fig. 2. The left part of the figure displays the results from RF models. For a more comprehensive analysis, we also show the best result obtained from the H_2_O AutoML model as shown in the right part of Fig. 2.

We acknowledge that binarization does lead to information loss. The continuous modeling results demonstrate that predicting Yield_Plant is particularly challenging, likely due to the biological variability between individual plants. However, we observed better performance for Yield_Meter, which is a more stable measure due to its aggregation over a larger area. To balance accuracy and complexity in this study, we applied binarization to the continuous response in cases where it improved performance, although we recognize that this is not an ideal long-term solution. In future work, we plan to explore larger datasets and incorporate additional environmental variables to enhance model accuracy without relying on binarization.

### Comparing the performance of random forest and H_2_O autoML for disease and binarized yield prediction

**Fig. 3:**
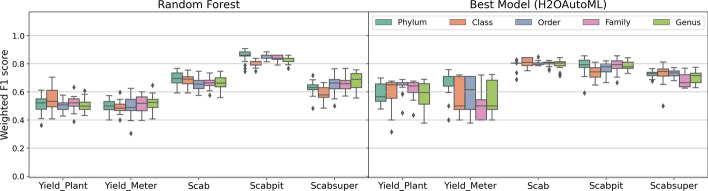
Boxplots weighted F1 score values: random forest and Best Model resulted from the H_2_OAutoML python package. The x-axis represents the diseases and yields outcomes. The range of each box plot depicts the weighted F1 score values for 20 normalized datasets at each taxonomic level

Figure 3 presents the weighted F1 scores for RF (left panel) and the best H_2_O AutoML models (right panel) across various responses, including both yield and disease outcomes (binary responses). Each boxplot represents different taxonomic levels (Phylum, Class, Order, Family, Genus) to evaluate model performance. The results show that RF performs comparable to the best H_2_O AutoML models, particularly excelling in Scabpit response. This demonstrates RF’s reliability, as its performance is consistently close to or equal to the more complex models selected by AutoML.

We also choose Deep Learning Models as they are known to excel in exploring deep relationships between predictors. We believe it is essential to include a Deep Learning model as this is the cutting-edge method that resulted in most success in ML applications in the last decade. However, while deep learning approaches were considered, we determined that our dataset, with approximately 200 samples, is too small to effectively apply deep learning models. Deep learning typically requires larger datasets to avoid overfitting and produce generalizable results, and thus, it was not a viable option for our study. While there are many computational efficient models, none would be properly trained with 200 samples without underfitting or overfitting. Despite the computational challenges, Bayesian NNs [13] are known to be informative for small sample sizes, offering protection against overfitting by modeling parameter uncertainty. Even though the Markov Chain Monte Carlo (MCMC) process for parameter estimation is very computationally inefficient in the context of Deep Learning, more research has gone into alternate estimation methods such as Variational Inference methods in the past few years which might drastically improve the computational efficiency in the near future. The Deep Learning result also validates the result of the RF model, a completely different approach that is more computationally efficient. Therefore, we will retain the Bayesian NN results in the paper alongside the other models, as they provide valuable insights despite the computational demands.

Regarding black scurf disease, we found that it is a very imbalanced dataset, with only 6 samples exhibiting the disease. Due to the extremely limited number of cases, the results from machine learning methods cannot be considered reliable. Consequently, we decided not to focus on this disease in our current analysis. Instead, we plan to use data augmentation methods to improve predictions for black scurf. Additionally, we are collecting more datasets, after which we will apply ML methods to the original data to obtain more reliable results.

Although the H_2_O AutoML framework identifies slightly better-performing models in certain cases, RF maintains a strong balance between predictive performance and computational efficiency. In yield-related predictions like Yield_Plant and Yield_Meter, RF produces F1 scores that are very close to those of the best AutoML models, making it a dependable choice. Given the practical constraints of running models across 600 configurations (5 taxonomic levels $\times$ 20 normalization methods $\times$ 6 responses) and the inclusion of environmental predictors, RF’s lower computational demand provides an efficient solution without compromising accuracy. This comparison supports our choice of RF as a reliable and efficient model, reinforcing the robustness of our study’s findings. Furthermore, we aimed to use one robust model that works effectively across all response types, and RF consistently met this criterion, demonstrating reliable performance across various conditions. Given these results, RF was selected as the main model due to its interpretability, robustness, and lower computational demands. While H_2_O AutoML occasionally yielded slightly higher scores, RF’s performance remained consistently close, underscoring its suitability as the primary model for microbiome studies with limited computational resources.

### Overall performance of predictive models: manual binarization causes inaccurate prediction of yield

**Fig. 4:**
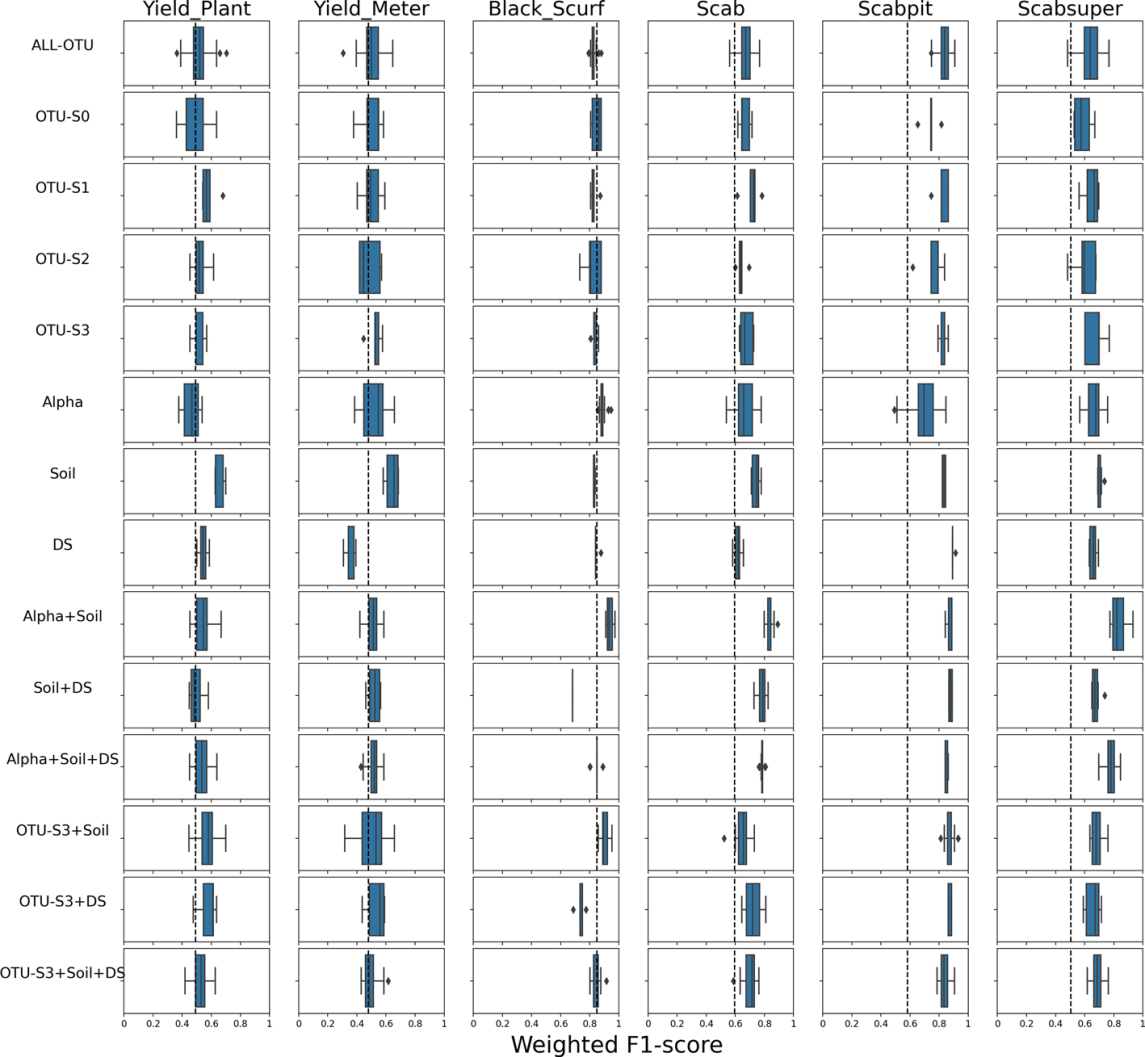
Boxplots of weighted F1 scores for random forest models for different types of predictors (rows) and different yield or disease outcomes (columns). For a description of the rows, see Table 2. The range of each boxplot depicts the weighted F1 scores for datasets in different taxonomic levels and different normalization and zero replacement strategies. The dashed line corresponds to the weighted F1 score when fitting the model with random datasets (see section B in the supplementary file)

First, we identify the outcomes (disease or binarized yield) that are accurately predicted across models (and are thus robust to prediction regardless of model choices), as well as the models that accurately predict across outcomes (and are thus the most powerful model alternatives). To do so, we aggregate the weighted F1 scores on data preprocessing choices such as normalization, zero replacement, and taxonomic levels for every model and every outcome. We employed RF and Bayesian NN models across various predictive scenarios (14 different models). The predictive capabilities of these models are illustrated in Figs. 4 (RF) and S3 (Bayesian NN), providing a comprehensive analysis of all 14 models outlined in Table 2. This detailed comparison illustrates how each feature selection strategy impacts model performance across taxonomic levels and response types. Columns correspond to the six responses: four diseases and two yield outcomes. For a given panel (model in row and response in column), the boxplot corresponds to the different weighted F1 scores for every combination of normalizations/zero replacement strategies as well as different taxonomic levels (Table S4). For example, the boxplots for the ALL-OTU model (first row) include weighted F1 scores of the model fit on 20 normalization/zero replacement strategies, and 5 taxonomic levels (100 different weighted F1 scores per outcome). The performance of these models was assessed using the weighted F1 score metric, which accounts for the imbalance in classes, making it particularly suited for this dataset. The dashed line in each panel corresponds to the average weighted F1 score of the model when fit with all random datasets (see section “Prediction of potato disease from microbiome data” and Figs. S6 and S7 in Supplementary file). This line allows us to assess whether the real data has more predictive power than random data [39]. For Random Forest models, feature selection performance ranks in this order: OTU-S3 > OTU-S1 > OTU-S2 > OTU-S0. Combining OTU-S3 with environmental information further enhances performance. Figure S3 follows the same structure for Bayesian NN models. Similar to Random Forest, OTU-S3 shows better performance compared to OTU-S1, OTU-S2, and OTU-S0. The best results are achieved by combining OTU-S3 with soil information. The comparison against random datasets provides a baseline, helping to ensure that the model’s performance is not due to chance but reflects real patterns in the data.

While these plots do not allow us to distinguish differences by taxonomic level or normalization/zero replacement strategy (more on that in the next subsections), we can identify outcomes (columns) that can be more accurately predicted across models (rows). Additionally, we can identify models that are capable of accurately predicting more outcomes. It is readily evident, for example, that the yield outcomes cannot be accurately predicted by any model as all the weighted F1 scores fall consistently below the dashed line. It is notable that the yield responses are those for which there is not a clear binarization strategy. Since we are artificially separating samples into the two classes (low and high yield) based on whether they are above or below the variety-specific median, samples on the boundary will in fact be very similar to each other, and thus, difficult to classify. Furthermore, the poor prediction of yield is not restricted to one data type (microbiome vs environmental) which also suggests that the prediction challenges arise from the binarization process rather than the model or set of predictors.

Disease outcomes, on the contrary, display higher weighted F1 scores overall, and in particular, pitted scab displays weighted F1 scores that are consistently above the random prediction dashed line across different models. For the case of black scurf disease, even when the weighted F1 scores are very high, this is a deceiving result, as this disease outcome is highly imbalanced. This means that a naive model predicting all samples to belong to the majority class will have high prediction accuracy (see dashed line above 0.8 for random data). We investigate the prediction of black scurf disease more carefully with the augmented data that balances the proportion of both classes (Section “Robust prediction in imbalanced datasets with data augmentation”).

**Fig. 5:**
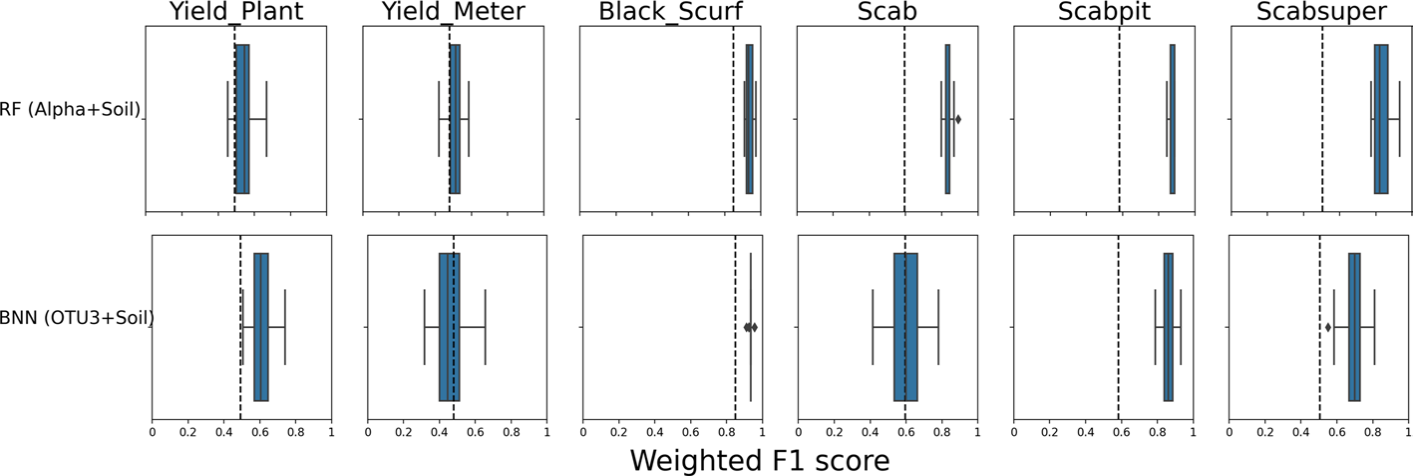
Boxplots represent the weighted F1 scores corresponding to the most accurate predictions achieved by RF and Bayesian NN across various yield or disease outcomes (columns). The RF model exhibits its highest accuracy when utilizing alpha diversity and soil chemistry data (Alpha+Soil), while the Bayesian NN models demonstrate optimal performance by integrating OTUs identified as important by both machine learning and network comparison strategies, along with soil chemistry data (OTU-S3+Soil). Each boxplot range depicts the weighted F1 scores for datasets at different taxonomic levels and normalization methods, with the dashed line indicating the score obtained from fitting the model with random datasets (see section B in Supplementary file). Detailed results are provided in Figs. 4 (RF) and S3 (Bayesian NN)

Figure 5 showcases the best performance of RF and Bayesian NN models. In the RF model, the integration of alpha diversity and soil chemistry data (referred to as Alpha+Soil) yields the most accurate predictions across all outcomes. Conversely, optimal performance for the Bayesian NN models is achieved by OTUs identified as significant by both ML and network comparison strategies (more details on feature selection are provided in section “Effective preservation of predictive signal with different feature selection strategies”), alongside soil chemistry data (denoted as OTU-S3+Soil). These findings highlight the importance of integrating environmental information with microbiome data to enhance predictive power for disease outcomes.

Finally, given the poor performance on yield, we focus on the fine-grained description of results for the disease outcomes only for the remaining of the manuscript.

### Prediction of potato disease from microbiome data

#### Normalization and zero replacement strategy has been proven to have impact on predictive power

**Fig. 6:**
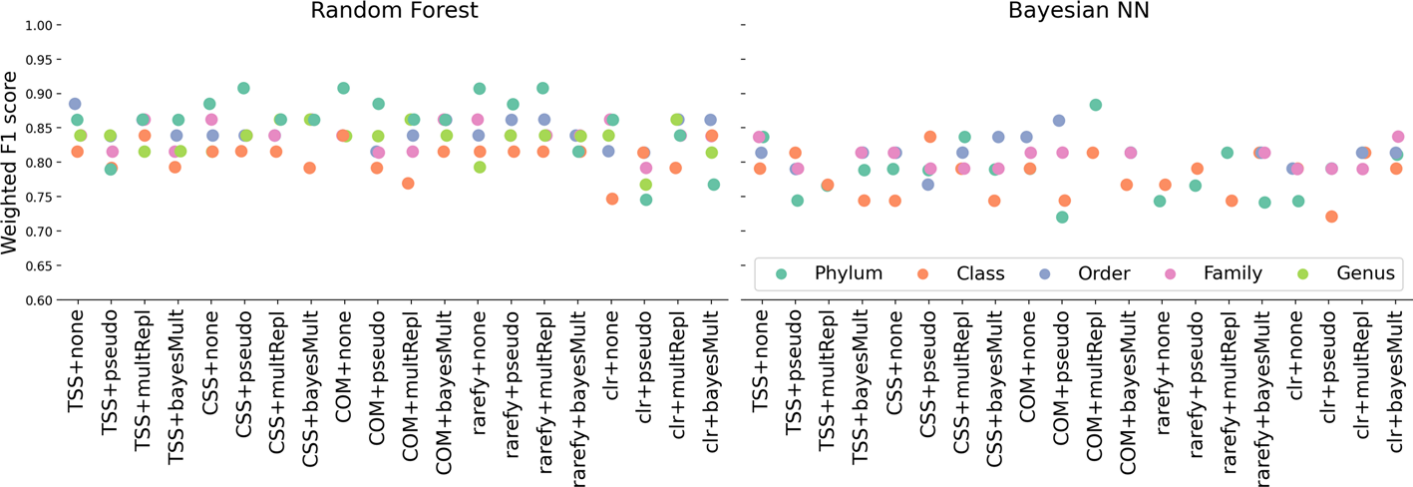
Weighted F1 scores (y-axis) for random forest and Bayesian neural network (Bayesian NN) models for the pitted scab disease under the 20 normalization/zero replacement strategies (x-axis). We can conclude, however, that taxonomic levels, normalization and zero replacement strategies have an effect on the prediction accuracy of the models as evidenced by the broad range displayed by the points

One of the goals of our study is to identify ideal data preprocessing steps that are guaranteed to maximize predictive power on the ML models. Figure 6 shows the weighted F1 scores for pitted scab for different combinations of normalization and zero replacement strategies (x-axis) for the two types of models (RF and Bayesian NN). These analyses include all OTUs under the five taxonomic levels (different colors). Similar plots for other diseases are presented in Figs. S8, S9, S10, S11 and S12 in the Supplementary Material. In fact, there is considerable interaction between the normalization/zero replacement method and the taxonomic level. For example, for the RF model, the best result is achieved with Phylum level and cumulative sum scaling normalization with pseudo-zero replacement strategy (CSS+pseudo) or common sum scaling normalization without any zero replacement strategy (COM+none). Additionally, the rarefy+none and rarefy+multRepl strategies demonstrate good performance. For Bayesian NN, however, the best results are achieved with the common sum scaling normalization with the multiplicative zero replacement (COM+multRepl) for the Phylum level (See Fig. 6).

For a given normalization/zero replacement strategy (x-axis), the variability in the scatterplot points indicates that taxonomic levels have an impact on the predictive power of the model. When we compare the range of weighted F1 scores across normalization and zero replacement strategies, we see that the effect of the strategy is not negligible. For example, at the Phylum level, the lowest weighted F1 score is around 0.75 for centered log-ratio normalization with pseudo-zero replacement strategy (clr+pseudo) to around 0.9 for cumulative sum scaling normalization with pseudo-zero replacement strategy (CSS+pseudo). This implies that for a given taxonomic level, the resulted weighted F1 score will be highly influenced by the normalization and zero replacement strategy. Traditionally, microbiome researchers use the total sum scaling normalization without any zero replacement strategy (TSS+none) on their data which has a range of 0.80–0.90 weighted F1 scores for the RF model (0.8–0.85 for the Bayesian NN model) depending on the taxonomic level.

The strong interaction effects of taxonomic level, normalization, and zero replacement strategy prevent us from making recommendations about the best data preprocessing practices that can be generalizable to other datasets. We conclude by suggesting data practitioners to consider trying a variety of appropriate normalization and zero replacement strategies instead of relying solely on one approach, but see section “Identifying general practices to predict potato disease from microbiome data using full model selection models” for more recommendations.

#### Effective preservation of predictive signal with different feature selection strategies

**Fig. 7:**
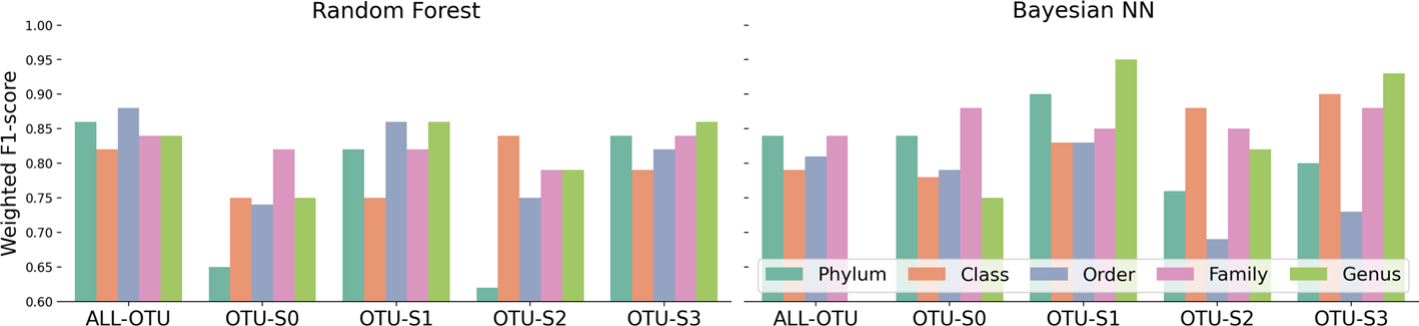
Weighted F1 scores (y-axis) by random forest and Bayesian neural network (Bayesian NN) models for the pitted scab disease (*Scabpit*) by feature selection strategy (x-axis) including all OTUs (All-OTU), OTUs selected by the ML method (OTU-S1), the network comparison method (OTU-S2), both methods (OTU-S3), or neither method (OTU-S0)

One of the standard steps in the ML pipeline is feature selection, especially for cases of high-dimensional data. We compare the ability to retain predictive signal of three feature selection strategies: standard importance score from ML methods, comparison of microbial network topologies, and combination of both. More details on the feature selection strategies can be found in Methods. Figure 7 shows the weighted F1 scores for the two types of models (RF and Bayesian NN) on pitted scab disease (Scapbit) under different subsets of predictors: (1) all OTUs (ALL-OTU), (2) only OTUs that were identified as important by the ML strategy (OTU-S1), (3) only OTUs that were identified as important by the network comparison strategy (OTU-S2), (4) OTUs that were identified as important by both strategies (OTU-S3), or (5) OTUs that were not identified as important by neither strategy (OTU-S0). For fair comparison, we include the same number of predictors in OTU-S0 as in OTU-S3. Similar figures for other responses are shown in Figs. S13, S14, S15, S16 and S17 in the Supplementary Material.

Again, we perceive a strong interaction between taxonomic level and feature selection strategy. For the RF model, the highest weighted F1 score is achieved when including all OTUs (ALL-OTU) at the Order level whereas for the Bayesian NN model, the highest weighted F1 score is achieved when including OTUs identified by the ML strategy (OTU-S1) at the Genus level. RF models on all OTUs (ALL-OTU) have a weighted F1 score above 0.8 in all taxonomic levels which suggests that this model could be a better alternative compared to Bayesian NN which is more computationally intensive. There are also smaller differences in RF models when comparing the performance on OTU-S3 (important OTUs) and ALL-OTU (all OTUs) which suggests that the feature selection strategy is sufficient to preserve the predictive signal in the data while reducing the number of predictors in the model. This is relevant for computationally intensive models such as Bayesian NN that do not allow the inclusion of all OTUs for certain taxonomic levels.

To provide more interpretability, we compiled a comprehensive table that lists the key taxa across different taxonomic levels and responses. Each taxon is assigned a score based on its selection by ML and network-based feature selection methods: 0: OTUs not selected by either ML-based or network-based feature selection. 1: OTUs selected by ML-based feature selection. 2: OTUs selected by network-based feature selection. 3: OTUs selected by both ML-based and network-based approaches. This scoring system identifies the microbial taxa with the highest predictive importance for disease suppression or yield outcomes. End-users can access the corresponding table on our GitHub repository (link: https://github.com/solislemuslab/soil-microbiome-nn/blob/master/python-code/important_features_score.xlsx) to determine which taxa are most relevant for practical interventions or microbiome management strategies in their fields. We focused on the top five taxa in each taxonomic level to examine the literature for evidence of their importance in soil microbiome studies. In each level, we found support for their key roles, which aligns with our findings, indicating that our methods for feature selection and combining the two approaches to identify reliable taxa were successful. Other important taxa, such as the top 10 percent, can be considered for further studies as significant candidates. For instance, our models showed that taxa from abundant phyla such as *Proteobacteria* and *Chloroflexi*, as well as taxa from less abundant phyla including *Myxococcota*, *Spirochaetota*, and *NB1.j*, were significant predictors (see Tables S5 and S6 in the Supplementary Materials). This suggests that both dominant and rare microbial community members play a crucial role in ecosystem functions related to crop health, such as nutrient cycling, growth promotion, and disease suppression [40, 41]. In addition, some of the key taxa identified have well-established roles in agricultural systems. For example, taxa from the Class level: *Alphaproteobacteria* and *Gammaproteobacteria* are frequently studied for their roles in soil health and disease suppression [42, 43]. Furthermore, *Paenibacillaceae* [44] and, *Syntrophaceae* [45] at the Family level have been recognized for their plant-growth-promoting properties and biocontrol capabilities. However, some taxa from the Genus level we identified, such as *Acidothermus* [46], *Myxococcota* [47], and *Haliangium* [48] are relatively novel in this context and could represent promising candidates for further research. Moreover, *Pseudonocardiales* [49] and *Frankiales* [50] were detected as important taxa at the Order level.

#### Robust prediction in imbalanced datasets with data augmentation

High prediction power in imbalanced datasets is misleading as a naive predictor that classifies all samples as the majority class will have high accuracy. In our data, black scurf disease is highly imbalanced, and thus, the high prediction accuracy is unreliable. We confirmed, however, that after data augmentation which balanced the data, accurate prediction persisted.

**Fig. 8:**
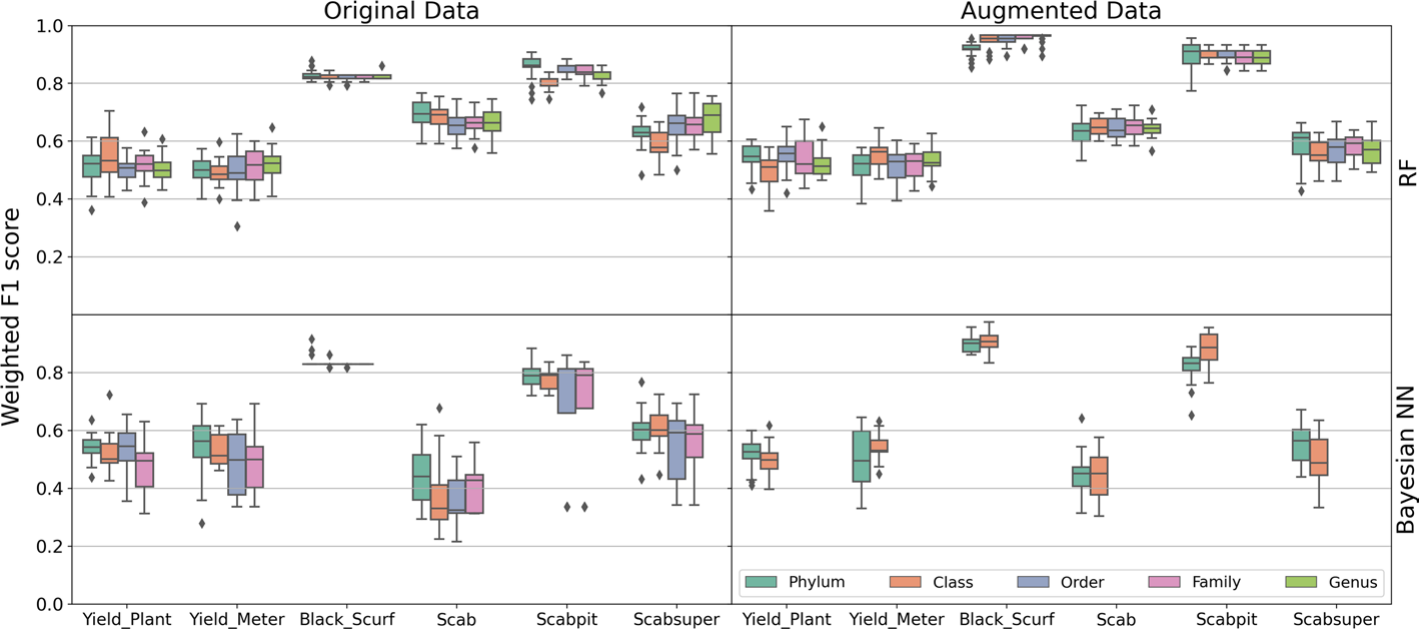
Boxplots with weighted F1 scores by model (rows): random forest (RF) and Bayesian neural network (Bayesian NN) for original and augmented OTU predictors (columns). The x-axis represents the yield and disease outcomes. The range of each box plot depicts the weighted F1 scores for 20 normalized datasets at each taxonomic level

To illustrate this, Fig. 8 depicts the weighted F1 scores on original and augmented datasets for all yield and disease outcomes (x-axis) and both models (RF and Bayesian NN). The range of each box plot depicts the weighted F1 scores for 20 normalized datasets at each taxonomic level. We observe that black scurf and pitted scab can be reliably predicted across taxonomic levels as their median weighted F1 scores for all taxonomic orders are around 0.8 when the models are fitted on the original datasets. As mentioned before, however, black scurf is highly imbalanced, so the results on the original data are not reliable. Fortunately, the median weighted F1 scores on augmented data (which is perfectly balanced by design) increase for both diseases, such that they are around 0.9 for all taxonomic levels. These results suggest that data augmentation, especially in cases of highly imbalanced data, is an appropriate strategy that improves the robustness of the model and, in some cases even increases the accuracy. One has to be careful, however, in that augmented data can yield certain models prohibited. For example, the Bayesian NN model could not be fit on the augmented datasets for Order, Family, or Genus levels due to computational limitations.

#### Identifying general practices to predict potato disease from microbiome data using full model selection models

As evidenced by our analyses, every single data and model choice has an impact on the predictive performance of our methods. The effects of different data preprocessing steps appear to strongly interact, and thus, we could not identify clear patterns on strategies to maximize prediction power.

**Fig. 9:**
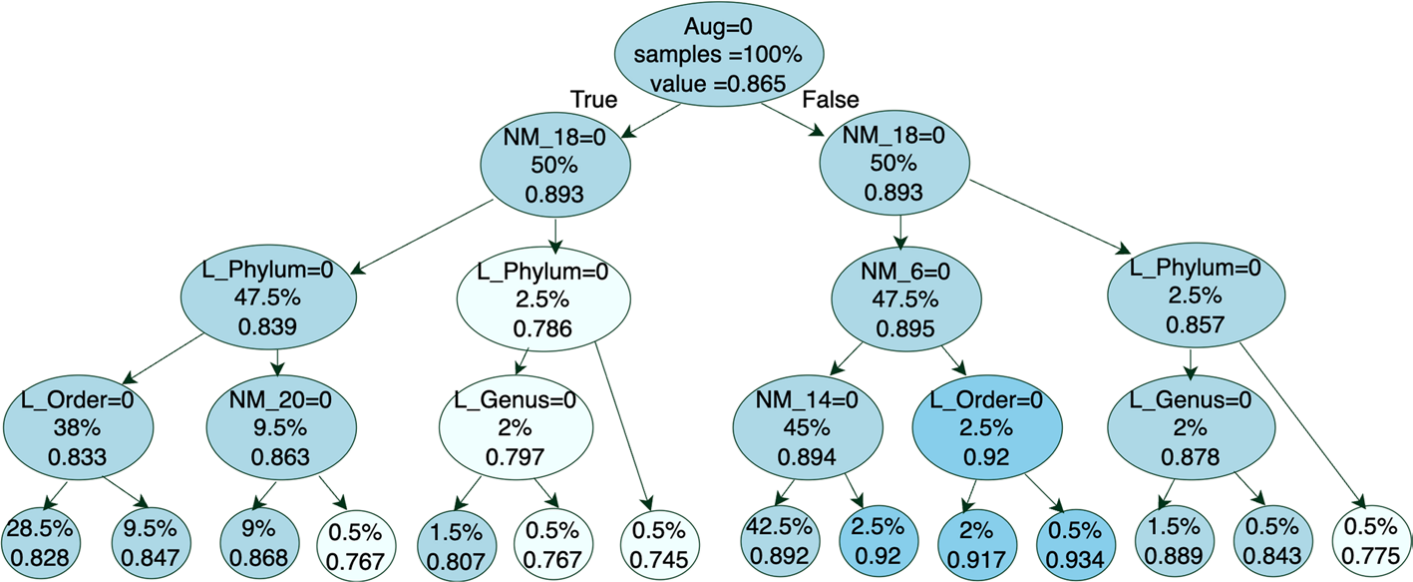
Full model selection decision tree with a maximum depth of 4 summarizing the results of random forest models on pitted scab disease. When the condition at a node is true, we follow the branch on the left, and when the condition is false, we follow the branch on the right. The percentage of the data preprocessing options and mean of weighted F1 scores are shown in each node

With a FMS [16, 17, 37] strategy, however, we are able to identify the choices that yield the highest measure of performance. More details on the FMS models can be found in Methods. Figures 9 and S23 show the FMS decision trees for the RF and Bayesian NN models on pitted scab disease, respectively. A FMS decision tree shows the different data preprocessing steps that yield different weighted F1 scores, so that practitioners can select the options that result in the highest predictive power. Here, we have five taxonomic levels, 20 normalization+zero replacement strategies, and 2 data augmentation options: no data augmentation (Aug=0) and data augmentation (Aug=1). Thus, in total, we have 200 data preprocessing options (20 normalization strategies times 5 taxonomic levels times 2 data augmentation).

To interpret a FMS decision tree, each node corresponds to a specific step in the data preprocessing pipeline, for example, whether to perform data augmentation or not. If the condition is true, we follow the branch to the left; if the condition is false, we follow the branch to the right. At the top of the decision tree, we have the root which represents the data preprocessing step that has the greatest effect on model accuracy. At the bottom of the decision tree, we have the leaves with the average weighted F1 score of the model fitted on the data that satisfies all conditions towards the root. Each node also displays the percentage of data preprocessing options included in the node. For example, in Fig. 9, the root node covers 100% of the options with average weighted F1 scores 0.865. The condition at the root node ($\text {Aug}=0$) represents the case of “no data augmentation”. Thus, “true” (left of the root) means “no data augmentation”, and “false” (right of the root) means “data augmentation”. For simplicity, we denote the 20 normalization/zero replacement strategies as $\text {NM}_i$ for $i=1,\dots ,20$. See section “Data filtering, normalization, and zero replacement” for a description on each normalization/zero replacement strategy.

For the FMS decision tree for the RF model (Fig. 9), the highest weighted F1 score (0.934 with 0.5% of the data) is achieved with data augmentation, normalization/zero replacement strategy #6 (CSS+pseudo), and Order level. Another path of the decision tree follows data augmentation and any normalization/zero replacement strategy except #6 (CSS+pseudo), #14 (rarefy+pseudo), and #18 (clr+pseudo) which yields an average weighted F1 scores of 0.892 for 42.5% of the data preprocessing options. If data augmentation is not an option (left of the root), the highest weighted F1 score available is 0.868 with Phylum level, and any normalization/zero replacement strategy except #18 (clr+pseudo) or #20 (clr+bayesMult). For the FMS decision trees on the other responses, see Figs. S18, S19, S20, S21 and S22 in the Supplementary Material.

Similarly, in Fig. S23 for the Bayesian NN model, the highest weighted F1 score (0.896 with 15% of the data preprocessing options) is achieved when we do data augmentation, we use any taxonomic level except Phylum, and we use any normalization/zero replacement strategy except #10 (COM+pseudo) and #18 (clr+pseudo). See Figs. S24, S25, S26, S27 and S28 in the Supplementary Material for other responses.

While the specific recommendations on normalization, zero replacement and taxonomic level are model-specific, both models perform better with data augmentation. In terms of taxonomic level, we note that the Bayesian NN was only run on Phylum, Class, and Family levels, and thus, the highest accuracy is obtained with Class level (when Phylum$=0$ is true). This does not contradict the result from the RF that identified Order level as the one yielding higher accuracy. We cannot rule out that the Bayesian NN would also have higher accuracy with Order compared to Class. The results from the RF, though, seem to suggest that there is a peak at Order, and more granularity in Family and Genus does not seem to provide more predictive power.

Table 3 presents a summary of the best FMS decision tree results from the RF model for all responses (Figs. 9, S18, S19, S20, S21 and S22). We focus on diseases that have reasonable outcomes. The most critical decisions across all diseases involve first utilizing the augmentation method and then selecting the appropriate taxonomic level-either Family or Order-while avoiding Phylum (due to its lower information content) and Genus (which can lead to overfitting due to small sample sizes). The final key factor influencing Random Forest results is the choice of the normalization method. Our analysis suggests the best results are achieved with NM6: CSS+pseudo and NM14: rarefy+pseudo, while the least effective methods were NM18: clr+pseudo, NM2: TSS+pseudo, and NM20: clr+bayesMult. We strongly recommend that future studies employ the FMS decision tree approach when a gold standard is available for evaluation. In cases where there is no way to find the optimal normalization method, we suggest applying multiple normalization strategies (as outlined in this paper) and reporting consensus results based on the outcomes of different normalized datasets. This approach can help yield more robust and reliable results.

**Table 3:** A summary of the best Full Model Selection (FMS) decision tree results from the Random Forest model. The table presents the preprocessing decisions for key disease outcomes and yield-related predictions. For each response, the most critical decisions-identified by their order in the FMS Tree (from the first to the fourth level, shown in the first to fourth columns)-such as augmentation methods, taxonomic levels, and normalization strategies, are shown. The corresponding weighted F1 scores and the percentage of data preprocessing options that follow this selection path are shown in the fifth and sixth columns, respectively

Response	Level 1	Level 2	Level 3	Level 4	F1-score	data preprocessing options (%)
Scabpit	Aug	Not NM_18	NM_6	order	0.934	0.50
Black_Scurf	Aug	Not Phylum	Not NM_18	Not NM_2	0.957	36
Scab	Not Aug	Not Phylum	Not NM_20	NM_14	0.71	2
Scabsuper	Not Aug	Not Genus	Family	NM_14	0.74	0.5
Yield_Plant	NM_2	Not Phylum	Not Order	Not Genus	0.623	2
Yield_Meter	Aug	Not NM_12	NM_9	Not Genus	0.575	2

For the Bayesian NN model, the focus was similarly on diseases with reasonable outcomes (Table 4). Unlike the RF model, we did not observe a consistent pattern regarding the importance of augmentation or taxonomic level (Figs. S23, S24, S25, S26, S27 and S28). However, the results indicate that the selection of augmentation, taxonomic level, and normalization methods at the first node of the FMS tree significantly influences the model’s performance. This variability could be attributed to computational limitations that prevented us from running the model on all taxonomic levels. The best-performing normalization methods for the BNN model were NM1: TSS+none, NM2: TSS+pseudo, NM4: TSS+bayesMult, NM7: CSS+multRepl, NM12: COM+bayesMult, NM13: rarefy+none, and NM16: rarefy+bayesMult.

In overall, with a deep investigation of the FMS results for all responses, we can recommend some normalization methods and taxonomic levels for further study. For RF model, it is recommended to use the augmenting method since RF can have better performance with more samples. For RF, normalization methods have a lower impact on the results, and in general, performing data augmentation and using a more specific taxonomic level like Class and Family are more important and located higher in the decision tree depth. This agrees with the fact that RF is known for being tolerant to high dimensional data, non-normal data, and missing values [51, 52]. In summary, four normalization methods consistently performed well across both disease and yield tasks: NM1: TSS+none, NM4: TSS+bayesMult, NM13: rarefy+none, and NM16: rarefy+bayesMult. There is no evidence from the FMS analysis to suggest that using these methods decreases performance, making them strong candidates for future studies.

**Table 4:** A summary of the best Full Model Selection (FMS) decision tree results from the Bayesian NN model. The table presents the preprocessing decisions for key disease outcomes and yield-related predictions. For each response, the most critical decisions-identified by their order in the FMS Tree (from the first to the fourth level, shown in the first to fourth columns). The corresponding weighted F1 scores and the percentage of data preprocessing options that follow this selection path are shown in the fifth and sixth columns, respectively

Response	Level 1	Level 2	Level 3	Level 4	F1-score	data preprocessing options (%)
Pitted scab disease	Aug	Phylum	Not NM_18	Not NM_6	0.834	15
black scurf diseas	Aug	Not NM_9	Not NM_8	Not NM_6	0.898	28.30
scab	phylum	Not NM_20	Not NM_5	Not NM_3	0.44	28.30
Scabsuper	Not NM_11	Not Aug	Not NM_15	Not NM_3	0.591	56.70
yield by plant	Not Family	Not NM_17	Not NM_14	Not NM_11	0.526	70.80
yield by meter	Not NM_10	Not NM_15	Not NM_19	Not Family	0.528	70.8

### Prediction of potato disease from environmental data

One of the questions to address in our work is whether prediction accuracy is improved by the inclusion of microbiome data, or if environmental factors (usually cheaper to collect) provide enough signal to classify potatoes in diseased or non-diseased groups. We found that environmental factors indeed provide sufficient signals to predict pitted scab disease as illustrated in Fig. S29 which shows the weighted F1 scores by RF and Bayesian NN models based on environmental (soil characteristics) data for pitted scab. The range of each boxplot corresponds to the six scaling methods described in section “Data filtering, normalization, and zero replacement”. In contrast with the normalization methods in microbiome data, we observe here that the scaling methods do not seem to have an effect on prediction as evidenced by narrow boxplots, and that weighted F1 scores are all higher than 0.75, and therefore, comparable to the models fitted on microbiome data alone. These results suggest that environmental factors alone are powerful to predict the incidence of pitted scab in the tubers. As microbiome data is more expensive than environmental data, we suggest to prefer environmental predictors under restricted monetary budget. See Figs. S30, S31, S32, S33 and S34 in the Supplementary Material for other responses.

### Leveraging microbiome and environmental data in the prediction of potato disease

**Fig. 10:**
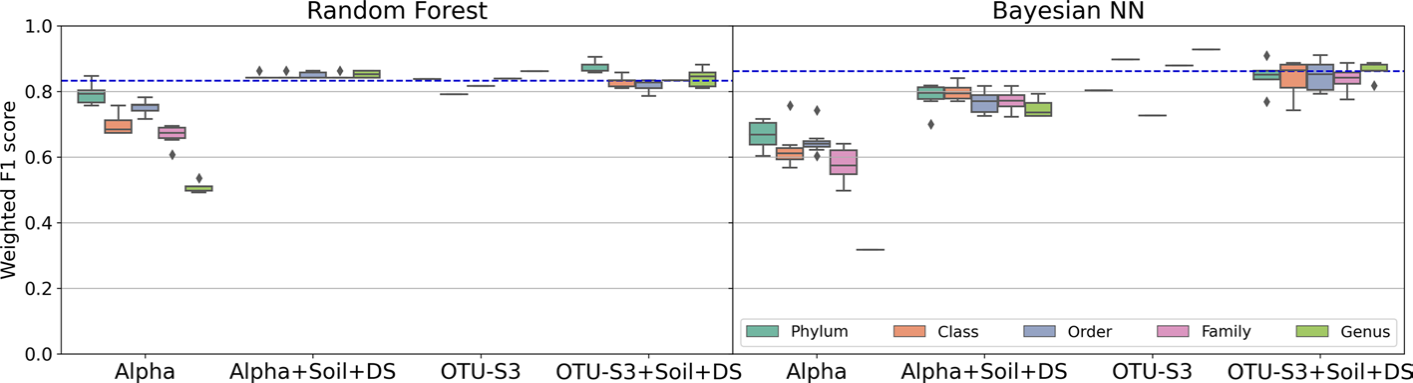
Boxplots with the weighted F1 scores (y-axis) by random forest and Bayesian neural network (Bayesian NN) models for pitted scab disease. The range of each box plot depicts the weighted F1 scores for normalized datasets at each taxonomic level. There are 6 normalization methods for alpha diversity and environmental factors (Soil and DS), and one normalization method for microbiome (OTU-S3). See Tables 2 and S4 for description of models and number of normalization methods for each predictors. The models including both types of predictors outperform other models, yet models including microbiome data alone (OTU-S3) are comparable which suggests that the microbial information indeed contains signal to predict the disease outcome on its own. However, microbiome data is more expensive to collect, and perhaps not necessary, given that the model without microbiome data (Soil) performs just as accurately (blue dashed line)

As expected, prediction accuracy improves when both microbiome and environmental data are included. Fig. 10 shows the weighted F1 scores by RF and Bayesian NN models based on combined datasets with environmental and microbial predictors for pitted scab. We only focus on the most accurate models identified in section “Overall performance of predictive models: Manual binarization causes inaccurate prediction of yield”. First, we note that a model that uses OTU abundances outperforms a model that uses alpha diversity as a predictor (comparison of Alpha with OTU-S3) for both types of models (RF and Bayesian NN). This suggests that we lose information by transforming abundances into diversity measures. Second, models including only OTU abundances (OTU-S3) perform comparably to models that include both types of predictors (OTU-S3+Soil+DS) which suggests that the microbial data indeed has substantial predictive power on its own, but adding microbiome to soil predictors may not provide much benefit for high predictive power, with the only exception of Phylum level OTU-S3+Soil+DS in a RF model (Fig. 10). Generally, the model with only soil information (shown as a blue dashed line) performs just as accurately. Third, contrary to prior expectations that microbial communities at finer resolution would be a better choice for predicting pitted scab or other diseases, our study does not find any evidence that the prediction power increases when moving up from Phylum to Genus level. Particularly, the prediction power of OTU-S3 in RF model increases from Class to Genus, and this pattern is not preserved when diversity is used instead of OTU abundances. For example, a model with only alpha diversity as the predictor (Alpha) shows decreasing weighted F1 score as we move from Phylum to Genus level. Both models (RF and Bayesian NN) when including all types of predictors (OTU-S3+Soil+DS) result in the similar weighted F1 score regardless of taxonomic level. See Figs. S35, S36, S37, S38 and S39 in the Supplementary Material for other responses.

According to Figs. 7, S13, S14, S15, S16 and S17 in the Supplementary Material, OTU-S3 performs well and is considered for comparison with other models in Figs. 10, S35, S36, S37, S38 and S39 in the Supplementary Material. By comparing results based on a few selected features (OTU-S3), we observe reliable performance for both disease and yield prediction. This underscores the robustness of OTU-S3 as an effective feature selection strategy.

### Running time

The RF model was implemented and tested on a MacBook Pro with an Apple M1 Pro chip and 16 GB of RAM. The running time for the RF model is provided in Table S8 in the supplementary materials. Depending on the number of trees and features selected, the RF model typically requires a few minutes per model on a dataset of our size (~200 samples). For the Bayesian NN model, the computational demands are significantly higher due to the need for probabilistic inference. We ran the Bayesian NN model on the Center for High Throughput Computation (CHTC) platform at UW-Madison with RTX2080ti graph cards. Despite using more computation resources, the Bayesian NN model would take between 24-72 h to compute the result for the models on all but Phylum level. Thus, it will be infeasible to run the Bayesian NN model on this particular problem on any personal devices. The runtime log of Bayesian NN models has been lost, unfortunately, as the CHTC platform only keeps the log files for 6 months, while the model was run more than 2 years ago. In summary, while the RF model can be efficiently run on a standard laptop such as a MacBook Pro, the Bayesian NN model requires significantly more computational resources. We recommend high-performance computing resources for readers planning to implement Bayesian NNs, particularly for larger datasets or more complex models.

## Discussion

The following points summarize the main findings and their significance in advancing our understanding of soil microbiome and plant health:

**Can machines classify what humans cannot? The importance of accurate labels.** The prediction power of microbiome data varies depending on the outcome we want to predict. For example, among all models that predict diseases, models for the pitted scab disease receive very high weighted F1 scores compared to other diseases. We further confirm this predictive power by comparing the performance of models trained on the real microbiome data to models trained on randomly generated data (see Figs. S6 and S7). Given that the prediction of the pitted scab disease is far from random, we can confidently conclude that this disease can be accurately predicted from microbiome data. It is noteworthy, however, that pitted scab disease is precisely one of the diseases that are easier to be visually detected, and thus, there is a reliable separation among the two classes (diseased and non-diseased) which is aiding in prediction by ML models. Other diseases, and more so yield, do not have such clear distinction between classes which results in lower predictive power. That is, we believe that the lack of prediction accuracy in yield is not driven by a lack of a biological connection between soil microbiome and yield, but on the lack of accurate labels that distinguish the two classes (e.g. low and high yield). If humans cannot distinguish what is low yield vs high yield, then that ambivalence will propagate into the ML classification. This conclusion seems to be confirmed when we notice that yield cannot be accurately predicted by any of 14 models in consideration (Table 2). We conclude that one of the main challenges when applying ML methods in biological applications is the artificial binarization of phenotypes. We acknowledge that binarization leads to information loss; however, in this study, we found that it improved model performance in some cases. Continuous modeling results indicated that predicting Yield_Plant is challenging due to individual plant variability, whereas Yield_Meter showed better performance as a more stable measure. In future work, we aim to explore larger datasets and additional environmental variables to enhance model accuracy without relying on binarization. More work is needed to improve the performance of regression models that can predict continuous phenotypes when faced with limited sample sizes that are common in biological domains.

**Human analytical choices: Data preprocessing has a substantial impact in prediction performance.** We demonstrate that the choice of normalization methods for microbiome datasets profoundly impacts prediction outcomes. Regrettably, our analysis did not reveal a discernible pattern indicating the superiority of one normalization method over others. We recommend domain scientists to explore various normalization methods for their data before utilizing them for prediction purposes. For further performance comparisons on normalization types, see also [27, 53, 54]. Upon comprehensive analysis of the FMS results, we offer recommendations for normalization methods for future investigations. For RF model, normalization methods have a lesser impact on results overall, with greater emphasis placed on other factors such as data augmentation and taxonomic levels. However, certain normalization methods are recommended against, as indicated in Table 3. In contrast, recommendations for the Bayesian NN model differ. Please refer to Table 4 for specific suggestions on normalization methods. In summary, four normalization methods consistently demonstrated strong performance across both disease and yield tasks for both RF and Bayesian NN: NM1 (TSS+none), NM4 (TSS+bayesMult), NM13 (rarefy+none), and NM16 (rarefy+bayesMult).

**Feature selection effectively preserves the predictive signal on lower dimensions.** In terms of feature selection, we considered different strategies to select important features (ML, network comparison, and intersection of both). There is a significant overlap between important OTUs by two methods and the inclusion of this subset of predictors allowed us to build less complex models with comparable good performance (see, for example, Fig. S7 for pitted scab disease). We did not observe one feature selection strategy that outperformed the others. For example, the weighted F1 scores obtained when using the OTUs identified as important by the ML methods are comparable to the weighted F1 scores when including OTUs identified as important by network comparison. It is worth mentioning, however, that the performance is also comparable to that obtained when including all OTUs which shows that the feature selection strategies work at preserving the OTUs that have predictive signals while simultaneously allowing computationally expensive models (like Bayesian NN) to be applied.

In addition to showing that both RF and Bayesian NN methods perform well in feature selection, we provide a list of important taxa identified through two different strategies in https://github.com/solislemuslab/soil-microbiome-nn/blob/master/python-code/important_features_score.xlsx. Our analysis shows that some of the microbial taxa might be important, such as *Paenibacillaceae*, *Moraxellaceae*, and *Syntrophaceae*, as they consistently proved to be key predictors in model performance. Those taxa may also be related to nutrient cycling, plant growth, and disease suppression [44, 47]. Important taxa with strong prediction power for yield and disease can play a significant role in sustainable agriculture, where maintaining healthy soil microbiomes is a key objective. These taxa can also inform future research on biofertilizer development and microbial inoculants in soil microbiome engineering. Our study provided a robust framework for identifying key microbial taxa using multiple classification methods, including RF and Bayesian NN.

**Finer taxonomic levels provide higher prediction power for Random Forest and not much for Bayesian NN.** While it is intuitive to expect that finer taxonomic levels would provide more predictive power, our analysis reveals this to be the case only in specific instances, such as the OTU-S3 model in RF and the OTU-S1 model in Bayesian NN, where the Genus level outperforms others (Fig. S7). However, this expectation does not hold true across all scenarios. This may be due to not having enough samples, which limits the predictive power at finer taxonomic levels. For a comprehensive assessment, we utilize the FMS method to identify optimal combinations of normalization, zero replacement, feature selection, and model choices for maximizing prediction accuracy in microbiome data analysis. Contrary to common expectations, our findings do not support an overall superiority of certain taxonomic levels over others. Instead, the FMS model provides nuanced recommendations: for RF, optimal results are achieved by employing data augmentation and focusing on more specific taxonomic levels such as Family and Genus, while for Bayesian NN, utilizing more general taxonomic levels like Phylum without augmentation proves advantageous.

**Limited predictive power in soil microbiome compared to environment.** When including environmental features such as soil physicochemical properties and microbial population density of soil in the model, we achieved higher weighted F1 score values. For pitted scab disease (*Scabpit*), utilizing alpha diversity with the RF model yields a median weighted F1 score of approximately 0.75. When combined with soil population density information, this score increases to 0.85. Similarly, for the Bayesian NN model, the score improves from 0.6 to 0.8 with the addition of soil population density information. For RF, employing OTU3 results in a median weighted F1 score of about 0.8, which further increases to 0.9 when supplemented with soil population density data. Similarly, for Bayesian NN, the median score reaches approximately 0.9. However, incorporating the population density of soil information leads to a narrower range for the box plot. In general, we investigated 14 different models for yield and disease prediction with different combinations of microbiome and environmental data. Results show poor performance in predicting yield across different models. The best results for pitted scab are achieved by combining alpha diversity and soil chemistry (Alpha+Soil) for RF and important OTUs and soil data (OTU-S3+Soil) for Bayesian NN. The median weighted F1 scores for predicting diseases range from 0.8 to 0.9 for RF and from 0.6 to 0.9 for Bayesian NN models (refer to Fig. 5). Although the best-performing models include microbiome predictors (Alpha and OTU-S3), it is important to note that the models without microbiome data are comparably powerful as those including microbiome data. Specifically, for the RF method, the F1 score exceeds 0.75 for pitted scabe disease (*Scabpit*) and for black scurf, and surpasses 0.6 for scab and superficial scab (*Scabsuper*) diseases. While the models trained with microbiome data alone show that microbiome can effectively predict pitted scab disease, the fact that models without this type of data continue to perform well provides evidence that microbiome data may not be necessary to achieve reasonable prediction. In fact, when the collection of microbiome data requires much higher cost investment, the increased prediction accuracy by including microbiome predictors may not be enough to justify the extra cost.

**Cost Analysis and Practical Applications:** The public price for soil chemistry analysis from a commercial lab can be $9.75 per sample. Adding the costs for microbial population density analysis, which is $20 per sample, brings the total cost of environmental data used in our analysis to approximately $29.75 per sample. Microbiome data collection, performed at the University of Wisconsin-Madison Biotechnology Center can cost $32 per sample. The goal of this research is to provide yield and disease predictions before planting and guide management decisions that would occur during the growing season. The value of improved prediction accuracy depends on how this tool is used. For instance, fields predicted to have high disease pressure would suggest the need for disease-control methods, such as fumigation or fungicide application. In this case, predictions with a certain threshold of accuracy (e.g.,>5%) would provide sufficient information to make disease-control decisions. Corresponding management actions would reduce disease risks, and the benefits would depend on factors such as the predicted disease level, potato cultivar, and market price. In another scenario, yield prediction can assist with selecting potato cultivars that align with the producer’s goals. The ultimate goal of our research is to help create a decision-making platform where growers can choose between various management options and perform benefit and risk analyses. Growers often aim to produce multiple potato cultivars for various markets, and soil microbiome composition and disease pressure often vary significantly among potato fields on the same farm. By selecting the most suitable cultivars based on the field-specific conditions, growers can optimize production and environmental outcomes, and meet the goal of precision agriculture.

**Inconsistent Predictive Performance Across Outcomes: **One of the key challenges identified in this study is the inconsistency in predictive performance across different outcomes, particularly between disease and yield. While our models demonstrate robust predictive power for diseases such as pitted scab and superficial scab, the results for yield outcomes were notably weaker. This discrepancy likely stems from the complex and multifactorial nature of yield responses, which are influenced by environmental factors, soil properties, farming practices, and other variables not fully captured in our current dataset. Moreover, disease outcomes such as pitted scab tend to have clearer biological indicators, making them easier to model, whereas yield is a more complex and continuous trait, further complicating prediction. Our results also underscore that traits difficult for humans to detect, such as yield potential based on subtle microbial interactions, are also inherently challenging for machine learning models to predict. Furthermore, the limited sample size in this study may have compounded these issues, particularly for yield outcomes, where larger datasets are required to capture the full range of influential factors. Moving forward, we are collecting more samples from different years and locations, which will enhance the diversity and robustness of the dataset. This will enable the development of more generalized and accurate models capable of addressing the variability in agricultural outcomes. Our findings here lay the groundwork for further research, with the ultimate goal of creating models that are both reliable and broadly applicable to diverse agricultural datasets.

**Implications for disease management.** Pitted scab is a severe form of potato common scab, a soil-borne disease that significantly reduces potato yield. The pitted scab is caused by the pathogenic *Streptomyces* spp, with symptoms of deep, dark lesions on the tuber surface. The disease is known to be sensitive to soil physicochemical properties including soil pH and moisture content [55]. Soil microbial communities can also be related to the severity of the disease in the plants, as suppressive soils with unique microbiomes often cause the pathogen to fail to establish in the plants [40]. The role of soil microbiome in influencing soil-borne disease has captured great research interests in both soil health and disease management. This study shows the close association of pitted scab with soil physicochemical properties when sampling across two different states. Although soil microbiome information contributed a small amount of prediction power to the prediction of this disease, the increased precision of prediction suggests the importance of soil microbiome in disease development. Particularly at finer spatial scales, soil microbiome may explain more of the disease variation among fields with similar physicochemical properties.

## Conclusion

Soil microbiome represents the most complex and least understood aspect of soil health. In this study, we use ML techniques such as random forest (RF) and Bayesian neural network (Bayesian NN) to determine whether soil microbial information has any predictive power for plant outcomes such as yield and disease. The RF method consistently demonstrates superior performance across all models, underscoring its effectiveness compared to the Bayesian NN method. In this study, Bayesian NN takes far longer to train due to weight sampling and approximation, especially on more refined taxonomic levels such as Order, Family, and Genus. Thus, we believe that based on the current dataset, RF is the preferred decision-making model with high prediction potential for disease outcomes when including a combination of microbiome and environmental predictors. Our results indicate that microbiome data helps predict potato disease, but the best accuracy comes from combining it with environmental data. Given that prediction with environmental factors alone was sufficiently powerful, it is uncertain whether the extra expense to sequence microbiome data is worth the cost.

**Future work.** In our study, we initially attempted to predict the continuous response variable for yield using various machine learning techniques, including RF regression. However, the inherent ambiguity and subjectivity introduced by artificial labeling of the continuous data posed significant challenges, leading to unsatisfactory prediction accuracy [56]. To overcome this limitation, we employed a binary classification approach, which proved to be more effective in capturing the underlying patterns and achieving better predictive performance. Although this approach involves some loss of granularity, it allowed us to focus on the broader trends and mitigate the impact of labeling subjectivity [57]. In addition, we applied the principal component analysis (PCA) method. However, in our specific case, PCA did not yield significant improvements in terms of dimensionality reduction or feature extraction. We hypothesize that this could be due to the inherent complexity and nonlinear relationships present in our data, which may not be effectively captured by linear transformations like PCA [58]. As part of future work, we aim to investigate advanced machine learning techniques that can handle the inherent ambiguity and subjectivity present in continuously labeled data. This could involve ensemble methods, deep learning approaches, or techniques specifically designed to handle noisy or subjective labels [59]. Additionally, we plan to utilize the MiNAA package [60] to detect alignment pairs of taxa in two constructed networks (healthy and diseased). By doing so, we can identify important taxa by comparing the two networks and confirming their significance using three different methods (ML-network-based models and applying Minaa). This work is based on data from one year, 2019. The goal is to find model traits that remain consistent year after year, making them applicable to other datasets. we plan to expand the dataset to include multiple growing seasons and diverse geographic locations, which will provide a more comprehensive representation of environmental and agronomic conditions. This expanded dataset will enable us to revisit continuous prediction methods with a larger and more diverse sample, aiming to improve accuracy by incorporating more sophisticated regression techniques and addressing variability across different regions. By leveraging a richer dataset, we hope to refine our models to capture the nuanced interactions influencing yield outcomes, ultimately developing more generalizable and robust predictive tools for precision agriculture.

## Supplementary Information

Supplementary file 1.

## Data Availability

The 16 S and ITS amplicon sequencing data associated with this study are publicly available at the NCBI Short Read Archive under the BioProject PRJNA1135141. All reproducible scripts are open source and publicly available at https://github.com/solislemuslab/soil-microbiome-nn.

## Abbreviations

ML: Machine learning
ITS: Internal transcribed spacer
OTUs: Operational taxonomic units
RF: Random forests
Bayesian NN: Bayesian neural network
FMS: Full model selection
ASV: Amplicon sequence variant
TSS: Total sum scaling
CSS: Cumulative sum scaling
COM: Common sum scaling
Rarefy: Rarefying (random samples without replacement)
CLR: Centered log-ratio
RFE: Recursive feature elimination
SPRING: Semi-parametric rank-based approach for inference in graphical
HMC: Hamiltonian (hybrid) monte carlo
Scapbit: Pitted scab disease
PCA: Principal component analysis
