# Supplementary material for: Human limits in machine learning: prediction of potato yield and disease using soil microbiome data
Source: BMC Bioinformatics. 2024 Nov 26;25:366. doi: 10.1186/s12859-024-05977-2 (PMC11600749; doi:10.1186/s12859-024-05977-2)
Supplement: Supplementary file 1 [file 12859_2024_5977_MOESM1_ESM.pdf]

SUPPLEMENTARY MATERIAL: HUMAN LIMITS IN MACHINE LEARNING: PREDICTION OF POTATO YIELD AND DISEASE USING SOIL MICROBIOME DATA

Rosa Aghdam*
Wisconsin Institute for Discovery
University of Wisconsin-Madison
Madison, WI

Xudong Tang*
Department of Statistics
Wisconsin Institute for Discovery
University of Wisconsin-Madison
Madison, WI

Shan Shan
Department of Plant Pathology
Wisconsin Institute for Discovery
University of Wisconsin-Madison
Madison, WI

Richard Lankau
Department of Plant Pathology
Wisconsin Institute for Discovery
University of Wisconsin-Madison
Madison, WI

Claudia Solís-Lemus†
Department of Plant Pathology
Wisconsin Institute for Discovery
University of Wisconsin-Madison
Madison, WI

List of Tables

1 Technical terms: Machine Learning and Statistical Terms . . . 9
2 Description of variables in Soil . . . 10
3 Number of OTUs per taxonomic level (first column) in the original data (second column) and after filtering out OTUs that do not appear in at least 15 samples (third column). . . . 10
4 Types of predictors per model, number of taxonomic levels, normalization/zero replacement strategies, and range/number of predictors for each type. . . . 10
5 Selected features with different Machine Learning methods . . . 11
6 List of OTUs in microbial networks constructed by `SPRING` for two classes of scabpit . . . 12
7 Parameters for Random Forest Model . . . 12
8 Running Time for Random Forest. . . . 12

List of Figures

1 Flowchart for binarizing the continuous yield response . . . 3
2 Flowchart for data augmentation algorithm . . . 4
3 Boxplots of weighted F1 scores for Bayesian neural network models for different types of predictors (rows) and different yield or disease outcomes (columns) . . . 5
4 Microbial networks at Phylum level for non-diseased (Label 0) and diseased (Label 1) samples for pitted scab (Scabpit). . . . 6
5 Heatmap with scoring of OTUs (Phylum level) as important for each response. . . . 6
6 Scatterplot for F1 scores for different randomization strategy . . . 7

*Joint first authors with equal contribution in alphabetical order
†Corresponding author: solislemus@wisc.edu

7 Scatterplot for F1 scores for real and 200 generated datasets 8
8 Weighted F1 scores for yield by plant under the 20 normalization/zero replacement strategies 8
9 Weighted F1 scores for yield by meter under the 20 normalization/zero replacement strategies 13
10 Weighted F1 scores for black scurf disease under the 20 normalization/zero replacement strategies . . 13
11 Weighted F1 scores for scab disease under the 20 normalization/zero replacement strategies 13
12 Weighted F1 scores for superficial scab (Scabsuper) disease under the 20 normalization/zero replacement strategies 14
13 Weighted F1 scores for yield by plant and selected features by different strategies 14
14 Weighted F1 scores for yield by meter and selected features by different strategies 14
15 Weighted F1 scores for black scurf and selected features by different strategies 14
16 Weighted F1 scores for scab disease and selected features by different strategies 15
17 Weighted F1 scores for superficial scab (Scabsuper) disease and selected features by different strategies 15
18 Full model selection decision tree summarizing the results of Random Forest models on yield by plant 15
19 Full model selection decision tree summarizing the results of Random Forest models on yield by meter 16
20 Full model selection decision tree summarizing the results of Random Forest models on black scurf . 16
21 Full model selection decision tree summarizing the results of Random Forest models on scab disease . 17
22 Full model selection decision tree summarizing the results of Random Forest models on superficial scab (Scabsuper) disease 17
23 Full model selection decision tree summarized the results of Bayesian neural network models on pitted scab disease 18
24 Full model selection decision tree summarizing the results of Bayesian Neural Network on yield plant 18
25 Full model selection decision tree summarizing the results of Bayesian Neural Network on yield by meter 19
26 Full model selection decision tree summarizing the results of Bayesian Neural Network on black scurf 19
27 Full model selection decision tree summarizing the results of Bayesian Neural Network on scab disease 20
28 Full model selection decision tree summarizing the results of Bayesian Neural Network on superficial scab (Scabsuper) disease 20
29 Boxplots of the weighted F1 scores by random forest (RF) and Bayesian neural network (Bayesian NN) models for pitted scab disease using environmental predictors (Soil: Soil chemistry, DS: Microbial population density in soil). 21
30 Boxplots of the weighted F1 scores for yield by plant using environmental predictors 21
31 Boxplots of the weighted F1 scores for yield by meter using environmental predictors 21
32 Boxplots of the weighted F1 scores for black scurf disease using environmental predictors 22
33 Boxplots of the weighted F1 scores for scab disease using environmental predictors 22
34 Boxplots of the weighted F1 scores for superficial scab (Scabsuper) disease using environmental predictors 22
35 Boxplots of the weighted F1 scores for yield per plant based on Alpha, Alpha+Soil+DS, Soil+DS, OTU-S3, and OTU-S3+Soil+DS predictors 23
36 Boxplots of the weighted F1 scores for yield per meter based on Alpha, Alpha+Soil+DS, Soil+DS, OTU-S3, and OTU-S3+Soil+DS predictors 23
37 Boxplots of the weighted F1 scores for black scurf disease based on Alpha, Alpha+Soil+DS, Soil+DS, OTU-S3, and OTU-S3+Soil+DS predictors 23
38 Boxplots of the weighted F1 scores for scab disease based on Alpha, Alpha+Soil+DS, Soil+DS, OTU-S3, and OTU-S3+Soil+DS predictors 24
39 Boxplots of the weighted F1 scores for superficial scab (Scabsuper) disease based on Alpha, Alpha+Soil+DS, Soil+DS, OTU-S3, and OTU-S3+Soil+DS predictors 24

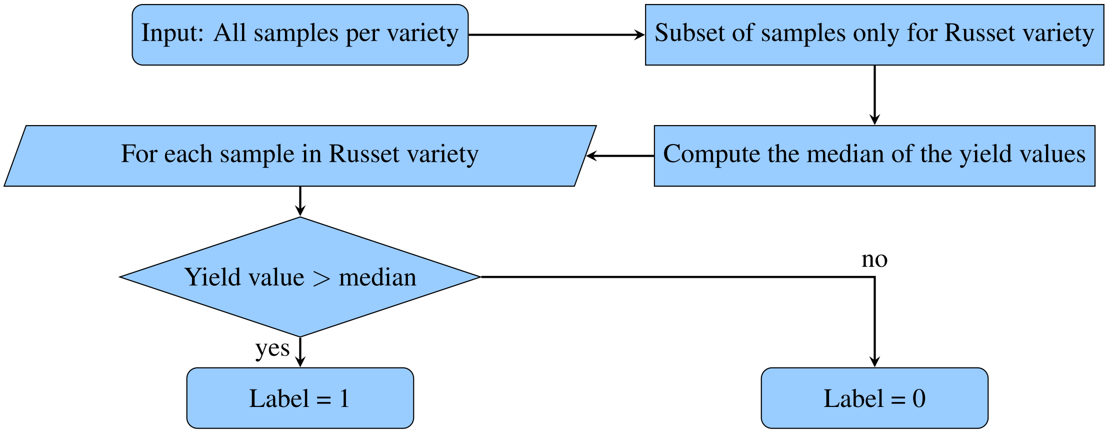

Figure 1: Flowchart for binarizing the continuous yield response into binary labels for the Russet variety. The same procedure is used for every variety in the dataset.

A Details of Bayesian Neural Network Model

We present the detailed description of Bayesian Neural Network here. Let $\mathcal{D} = \{x_i, y_i\}_{i=1}^n$ be the data with n samples where x_i is a N-dimensional feature vector in $\mathbb{R}^N$ and $y_i \in \mathbb{R}$ is the target response. A Bayesian NN model has L layers with K_l neurons in the lth layer. The weight parameters are defined in a set of matrices $\mathcal{W} = \{W_l\}_{l=1}^L$ where each W_l corresponds to the weights for the lth layer, and is of size $(K_{l-1}+1) \times K_l$. For an input $x_i \in \mathbb{R}^N$, the neural network maps it to a response $f(\mathcal{W}, \mathbf{x})$ by multiplying the input of each layer by the weights and then transforming via an activation function such as rectified linear unit (ReLU): $h(a) = \max(0, a)$. Unlike regular neural network models, a Bayesian NN imposes a prior distribution on the weights $\mathcal{W} \sim p(\mathcal{W})$ to capture their uncertainty. The posterior distribution of the weights given the data is then given by:

$$p(\mathcal{W}|\mathcal{D}) = \frac{p(\mathcal{W})\prod_{i=1}^n p(y_i|f(\mathcal{W}, x_i))}{p(\mathcal{D})}$$

, which can be used to predict the response **y*** of unseen data **x*** via

$$p(\mathbf{y*}|\mathbf{x*}) = \int p(\mathbf{y*}|f(\mathcal{W}, \mathbf{x*}))p(\mathcal{W}|\mathcal{D})d\mathcal{W}.$$

We use the original implementation of Bayesian NNs denoted "software for flexible Bayesian modeling and Markov chain sampling" for training and testing on our data. For each Bayesian NN model, we use five hidden layers, one input layer and one output layer. The input layer has n input neurons with n being the number of predictors for the model. This number varies depending on the predictors to include. For example, for the ALL-OTU model for the Phylum level, we have 42 predictors (Table 3), and thus the input layer has $N = 42$ neurons. For each hidden layer, we have $3N$ hidden neurons and one bias node. For the output layer, we only have one neuron with a binary value for the response. We use the hyperbolic tangent as the activation function: $f(x) = tanh(x)$ as opposed to the sigmoid function, also built in the software, as we found in practice that tanh performs much better.

We choose a hierarchical zero-mean Gaussian prior on all weights and on bias nodes. The prior for the weight from node i of one layer to node j of another layer is formulated as follows:

$$\mathcal{P}(w_{ij}|\sigma_{w_i}, \sigma_{a_j}) = \frac{1}{\sqrt{2\pi}\sigma_{w_i}\sigma_{a_j}}\exp(-\frac{w_{ij}^2}{2\sigma_{w_i}^2\sigma_{a_j}^2})$$

, where σ_{w_i} are the hyperparameters for the standard deviation of the priors for node i of layer w, and σ_{a_j} is the hyper parameter for the standard deviation of the priors for node j of layer a. Let $\tau_{w_i} = \sigma_{w_i}^{-2}$, then the hyper prior is given by

$$\mathcal{P}(\tau_{w_i}|\tau_w) = \frac{(\alpha_{w_i}/2\tau_w)^{\alpha_{w_i}/2-1}}{\Gamma(\alpha_{w_i}/2)}\tau_{w_i}^{\alpha_{w_i}/2-1}\exp(-\frac{\tau_{w_i}\alpha_{w_i}}{2\tau_w})$$

where α_{w_i} is the shape hyper parameter, and τ_w is the scale hyper parameter. For weights between hidden layers and bias nodes, we set $\alpha_{w_i} = 2$ and $\tau_w = 0.5$. For weights from the input layer to the first hidden layer, we set a prior

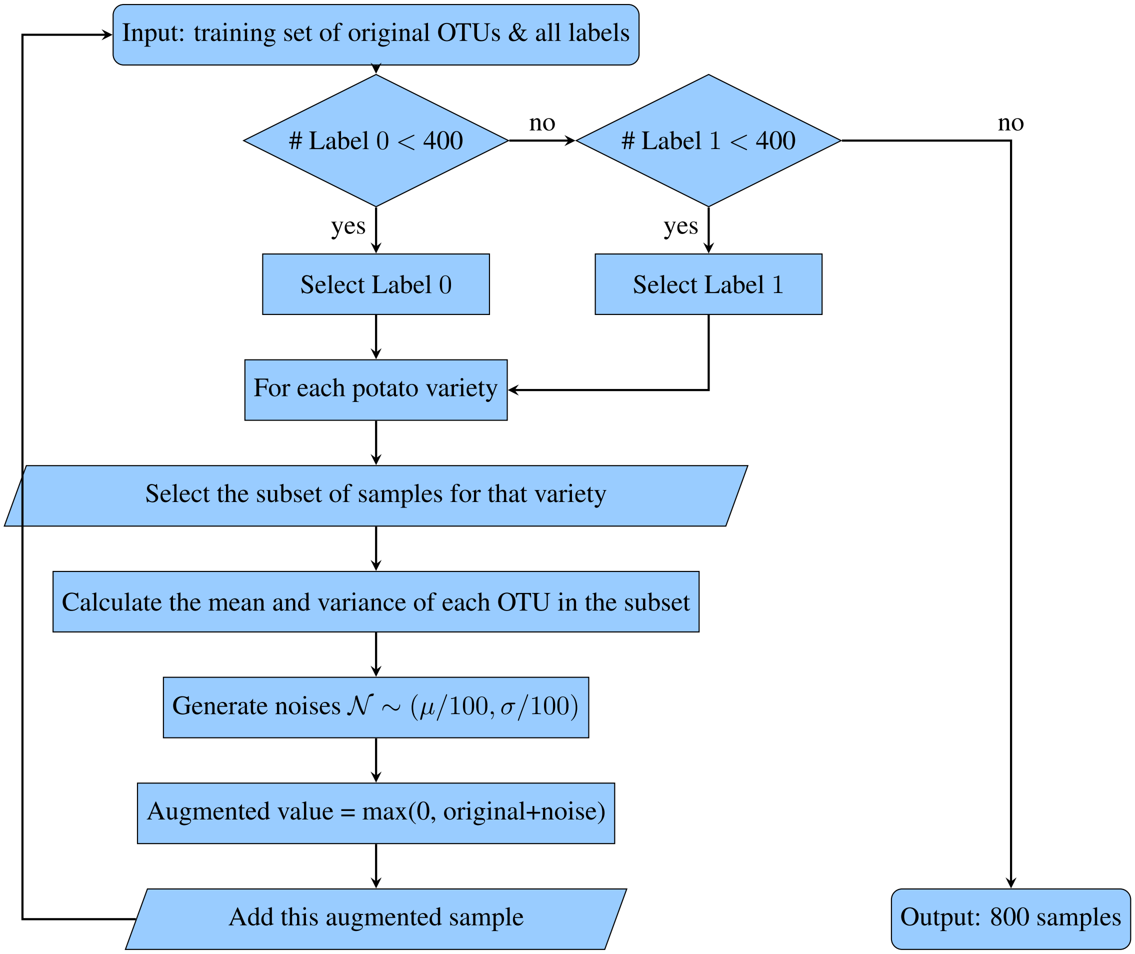

Figure 2: Flowchart with the data augmentation algorithm. The target sample size for the training set is 800 with 400 samples for each label. The noise that we artificially generate needs to be variety-specific before adding to the original samples so that the biological implications of the original samples would be preserved.

for τ_w as an inverse gamma distribution with shape hyper parameter $\alpha = 2$ and scale hyper parameter $\tau = 0.5$. This extra hyper parameter diminishes the effect of input nodes that have insufficient predictive power by setting weights arbitrarily close to zero.

The training and the approximation of the posterior distribution are done via a Hamiltonian (Hybrid) Monte Carlo (HMC) implemented in "software for flexible Bayesian modeling and Markov chain sampling". We select different leap frog lengths and step sizes for different models. Leapfrog lengths begin at 100 as starting point and are gradually decreased as a leapfrog length of 100 is expected to deal optimally with challenging estimation problems. Step size, on the other hand, is set at 0.1 as a starting point and it is decreased and increased in search for an average rejection rate smaller than 0.3. Burn-in is selected as the first half of the chain so that weights are sampled on the second half of the converged chain.

B More power to predict pitted scab disease in microbiome data compared to random data

In order to study whether the microbiome data has predictive signal to classify samples corresponding to diseased or non-diseased potatoes, we can compare the performance of our models when fitted on the real data and when fitted on completely random data. Intuitively, if the data has predictive signal, models fitted on real data will dramatically outperform models fitted on random data.

Figure 6 shows the scatterplot of F1 scores in the samples in one class (non-diseased potato: Label 0) versus the F1 scores in the samples in the other class (diseased potato: Label 1) for two disease outcomes (rows) and the four strategies

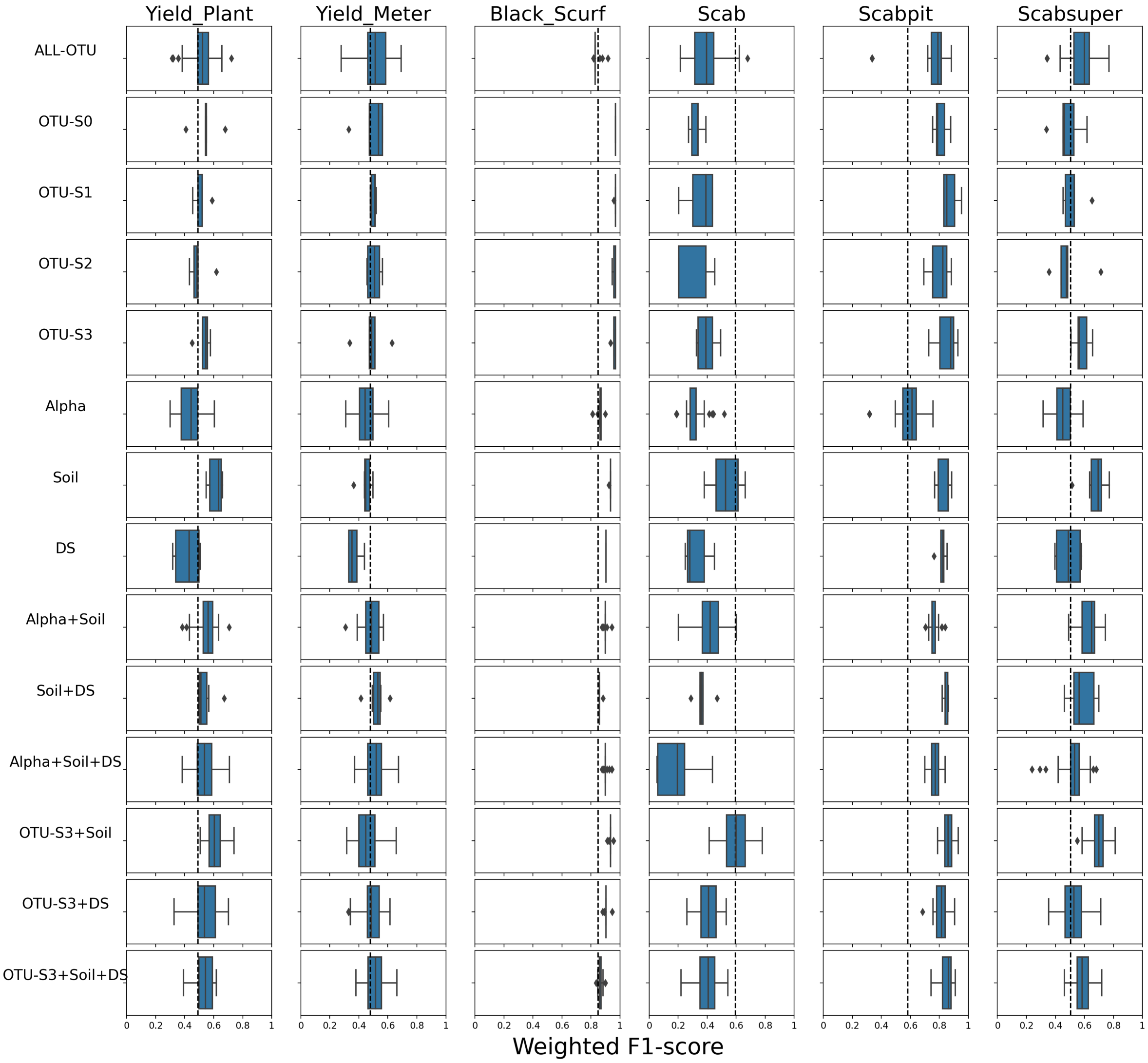

Figure 3: Boxplots of weighted F1 scores for Bayesian neural network models for different types of predictors (rows) and different yield or disease outcomes (columns). For description of the rows, see Table 2 in the main text. The range of each boxplot depicts the weighted F1 scores for datasets in different taxonomic levels and different normalization and zero replacement strategies. The dashed line corresponds to the weighted F1 score when fitting the model with random datasets (see Section B).

to generate random data (columns). Strategies correspond to randomization of rows or columns, or generation of random abundance values (more details on the randomization strategies in Methods). Each blue point in this scatterplot corresponds a random dataset, and the red point corresponds to the F1 scores when the model is fitted with the real data. Here, we only focus on microbiome data at the Phylum level.

For the pitted scab on the top row, the F1 scores for the original dataset (red dots) are on the upper right quadrant compared to points from random datasets (blue dots). This means that the F1 scores on the original data are higher than F1 scores on random data, and this, the original data has more predictive power than random data. However, for the superficial scab on the bottom row, many random datasets show better performance (higher F1 score values) than the real data, and thus, the real data does not contain much information to predict this disease. Other diseases as well as yield outcomes also underperform compared to random data as shown in Figure 7 in the Supplementary Material. We highlight that pitted scab is the most severe case of the potato disease common scab, and possibly a stronger signal of the soil microbial community composition and corresponding disease-suppressiveness than other measures of the disease such as superficial scab.

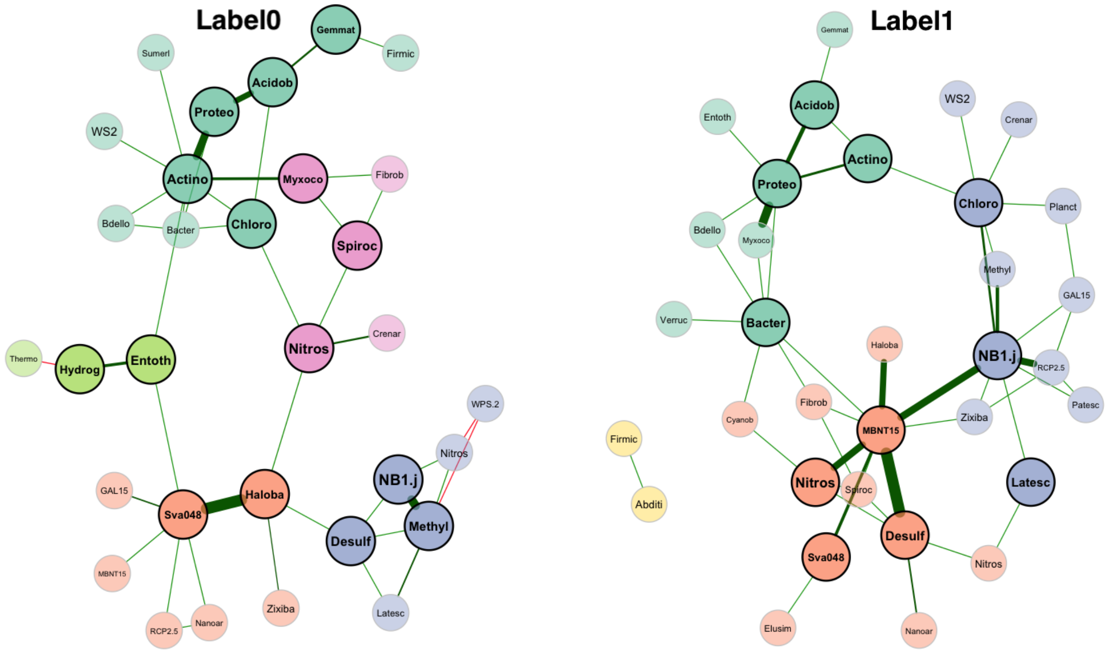

Figure 4: Microbial networks at Phylum level for non-diseased (Label 0) and diseased (Label 1) samples for pitted scab (Scabpit). We use the SPRING method [33] to reconstruct these microbial networks. Node colors represent clusters, which are determined using greedy modularity optimization. Clusters have the same color in both networks if they share at least two OTUs. Green edges correspond to positive associations and red edges to negative ones. Nodes that are unconnected in both groups are removed.

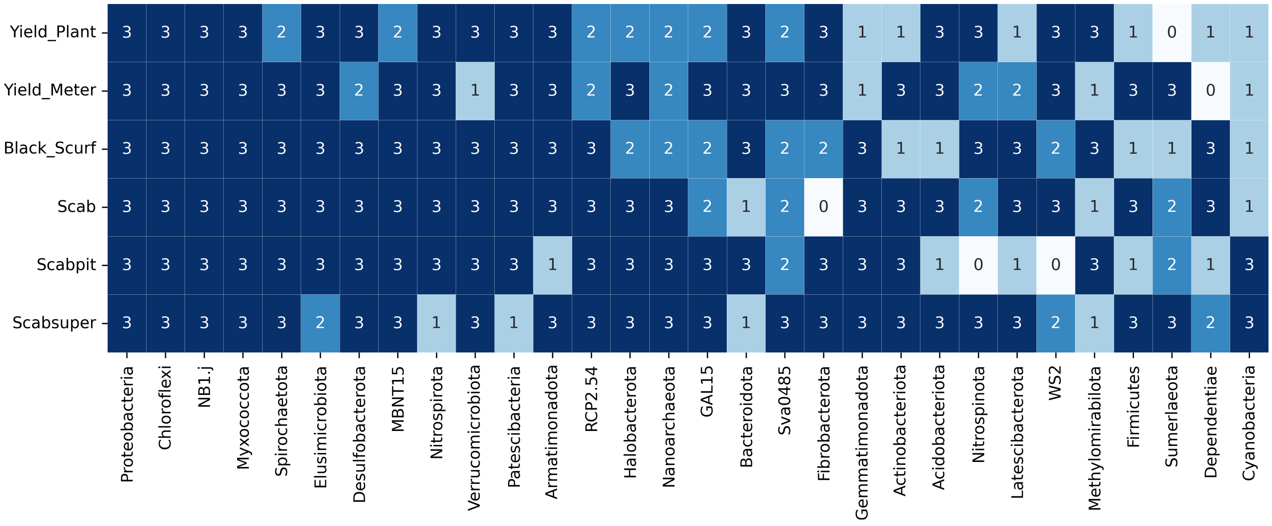

Figure 5: Heatmap with scoring of OTUs (Phylum level) as important for each response. Y-axis shows the responses and x-axis shows the OTUs in Phylum level. We define a scoring value for each OTU based on the selection by ML strategy ($score = 1$), network comparison strategy ($score = 2$), both ($score = 3$) or neither ($score = 0$).

B.1 Predictive Power of Microbiome Data: Comparison to Random Data

One of the key questions we want to address in this study is whether the microbiome data has predictive power to infer disease or yield outcomes. To address this question more formally, we devise a simulation scheme in which we simulate random microbiome data to train our RF model and compare the prediction potential with the trained RF model on real

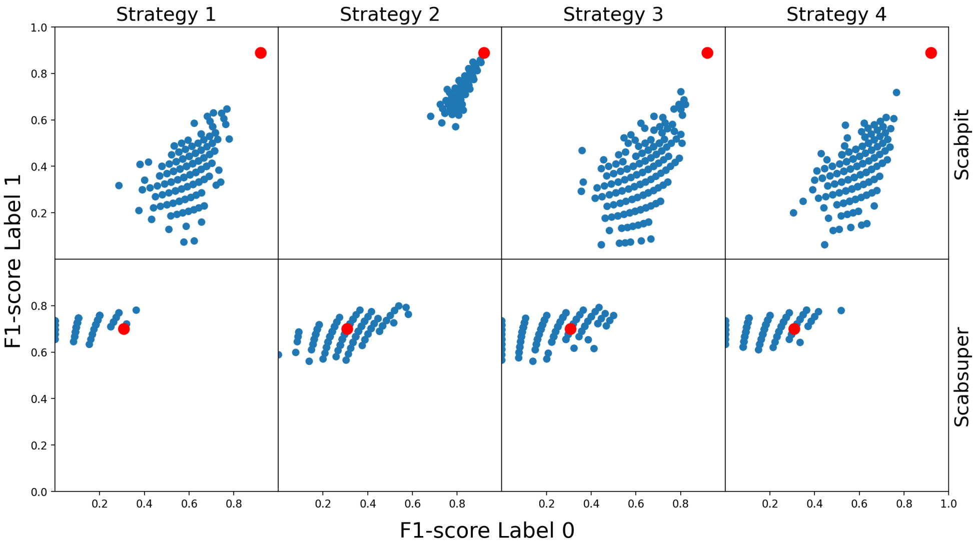

Figure 6: Scatterplot for F1 scores in the non-diseased (Label 0) group versus the F1 scores in the diseased (Label 1) group by disease outcome (rows) and randomization strategy (columns). Each panel has 200 generated datasets (each blue point is a random dataset). The red point corresponds to the F1 scores when the model is fitted on the real data. These results are for OTUs at the Phylum level. There is clear predictive power for the pitted scab (Scabpit) as the red dot is in the upper right region of the panel, but none for the superficial scab (Scabsuper).

microbiome data. If there is indeed a signal to predict yield or disease outcomes within the real data, then we expect the model trained with the real data to dramatically outperform the model trained with random microbiome datasets. We use four strategies to generate random datasets:

Strategy 1: Random matrix with values between 0 and 1, normalized so that each row has a sum of 1. We use the response vector from the original dataset.

Strategy 2: Real microbiome matrix where each entry that is greater than zero is replaced by a random number between 0 and 1. This strategy preserves the sparsity in the real data as zero entries remain zero. Rows are normalized so that the sum equals 1. We use the response vector from the original dataset.

Strategy 3: Real microbiome matrix is used with a permuted response vector.

Strategy 4: Rows in the real microbiome matrix are permuted, and the response vector is unchanged.

For each strategy, we generate $N = 200$ random datasets to train the RF model. Let F_i for $i = 1, ..., N$, denote the weighted F1 scores for the randomized data and let $F_{original}$ be the weighted F1 score on the real data. Let $X = \sum_{i=1}^{N} \mathbb{I}(F_i > F_{original})$ be the number of random datasets that perform better than the read data. We use the exceeding value (EV) $EV = \frac{X}{N}$ as test statistic to test the null hypothesis that the real data performs just as random data in the prediction on disease outcomes. The EV denotes the percentage of random datasets that perform better than the real microbiome data out of N random datasets. If $EV < \alpha$, we reject the null hypothesis and conclude that there is predictive power (beyond random) on the real microbiome data. Here, we set $\alpha = 0.05$.

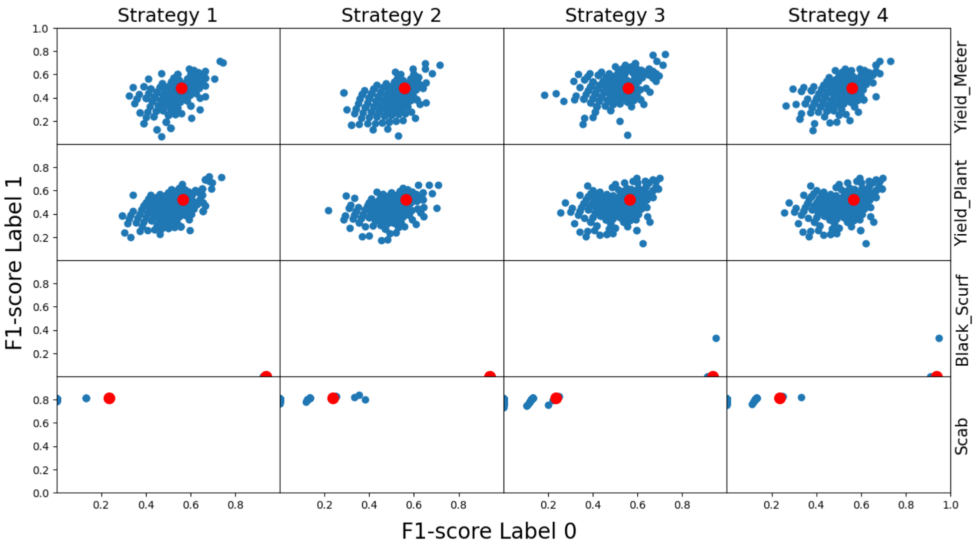

Figure 7: Scatterplot for F1 scores in the non-diseased (Label 0) group versus the F1 scores in the diseased (Label 1) group by disease outcome (rows) and randomization strategy (columns). Each panel has 200 generated datasets (each blue point is a random dataset), and in a red point, we should the results on the real data. These results are for data at the Phylum level. Fewer points in scab and black scurf diseases are due to extreme imbalance. For example, there are only 11 samples without disease (Label 0) out of 46 testing samples in Scab which results in 11 label 0 samples and only 12 possible F1 values for both 0 and 1 classes, i.e, only 12 points. Since there are far more label 1 samples in Scab, the model is biased towards label 1 and tend to predict most testing samples as label 1, so the F1 values are clustered around 75-85% for label 1. In this case, the points are more likely to stack on top of one another, thus leading to fewer points in the visualization.

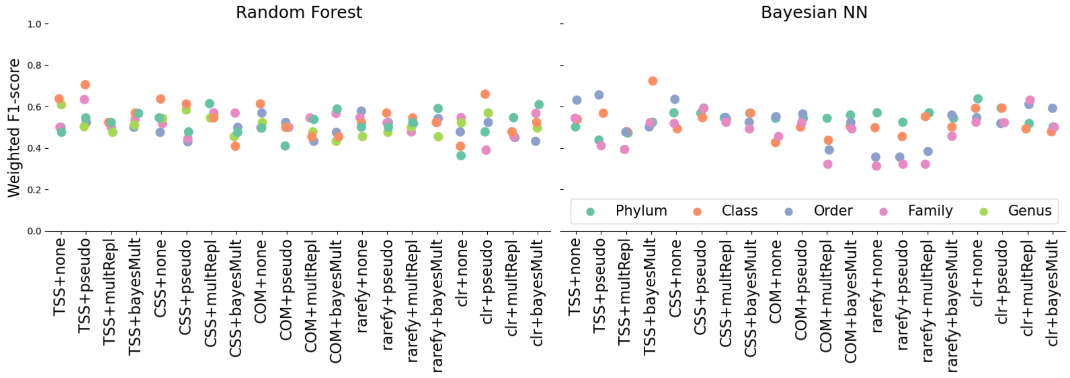

Figure 8: Weighted F1 scores (y-axis) for Random Forest and Bayesian Neural Network (NN) models for yield by plant under the 20 normalization/zero replacement strategies (x-axis). The lack of pattern prevents us from making recommendations of optimal strategies for microbiome OTU data. We can conclude, however, that taxonomic levels, normalization and zero replacement strategies have an effect on the prediction accuracy of the models as evidenced by the broad range displayed by the points.

Table 1: Technical terms: Machine Learning and Statistical Terms

Term	Definition
Alpha Diversity	A measure of the diversity within a particular area or ecosystem, typically expressed as the number of species (species richness) and their relative abundance in that ecosystem.
Beta Diversity	The ratio between regional and local species diversity, which measures the change in species composition between different ecosystems or along environmental gradients.
Taxonomic Levels	The hierarchical categories used in biological classification, such as Kingdom, Phylum, Class, Order, Family, Genus, and Species, with each level representing a more specific grouping of organisms.
Operational Taxonomic Units (OTUs)	In microbiology, OTUs are clusters of organisms grouped by DNA sequence similarity of a specific taxonomic marker gene. In other words, OTUs are pragmatic proxies for microbial "species" at different taxonomic levels.
Internal Transcribed Spacer (ITS)	A piece of non-functional RNA situated between structural ribosomal RNAs (rRNA) on a common precursor transcript. It is widely used in taxonomy and molecular phylogeny.
Amplicon Sequence Variant (ASV)	A unique DNA sequence recovered from a high-throughput marker gene analysis. Unlike OTUs, ASVs are exact sequence variants, providing a higher resolution approach to describing microbial community composition.
Machine Learning (ML)	A subfield of artificial intelligence focused on developing algorithms that enable systems to improve performance on tasks through experience without being explicitly programmed.
Random Forest (RF)	An ensemble learning method that constructs multiple decision trees and outputs the class that is the mode of the classes (for classification) or the mean prediction (for regression).
Bayesian Neural Network (Bayesian NN)	A neural network with a prior distribution on its weights, allowing for the quantification of uncertainties in predictions and helping prevent overfitting.
Principal Component Analysis (PCA)	A statistical procedure that uses orthogonal transformation to convert correlated variables into a set of linearly uncorrelated variables, called principal components.
Total Sum Scaling (TSS)	A normalization method where each value in a sample is divided by the total sum of all values, resulting in relative abundances.
Cumulative Sum Scaling (CSS)	A normalization method for microbiome data that corrects for varying sequencing depths by normalizing the sum of counts up to a particular quantile for each sample.
Centered Log-ratio (CLR) Transformation	A transformation for compositional data, involving the logarithm of the ratio of each component to the geometric mean of all components, making the data suitable for statistical analyses.
Recursive Feature Elimination (RFE)	A feature selection method that recursively prunes the least important features based on the performance of an estimator.
Semi-parametric Rank-based Approach for Inference in Graphical Models (SPRING)	A statistical method for inferring graphical models from high-dimensional data, particularly useful for microbiome datasets.
Hamiltonian Monte Carlo (HMC)	An algorithm for obtaining random samples from a probability distribution using Hamiltonian dynamics, allowing more efficient exploration of the distribution.
Compositionality	A property of data representing parts of a whole, typically expressed as proportions or percentages, where individual components are meaningful only in relation to other components.
Data Augmentation	Techniques used to increase the amount and diversity of training data without collecting new data, through transformations like geometric and color space augmentation.
Feature Selection	The process of selecting a subset of relevant features for model construction to improve interpretability and reduce overfitting.
Overfitting	A modeling error where the model captures noise and performs well on training data but poorly on new data, due to its inability to generalize.

Soil health	
Physicochemical (Soil)	pH<br>Texture<br>Organic matter content<br>Cation/Anion exchange capacity<br>Carbon fractions<br>Organic nitrogen<br>Available macronutrients<br>Available micronutrients<br>Potential carbon mineralization rate
Microbiome (OTU and alpha diversity)	Bacterial community composition, structure, diversity<br>Fungal community composition, structure, diversity<br>Bacterial and fungal population abundance
Pathogen	Verticillium dahliae, Pathogenic Streptomyces population<br>Disease suppressiveness (DS)<br>lab-estimated soil disease suppressiveness
**Potato crop assessment**	
Yield<br>Disease severity	Tuber Yield, yield by size class<br><br>Verticillium dahlia incidence<br>Common scab disease incidence and severity<br>Other diseases including hollow heart, silver scurf and black scurf
**Management practices**	
Field history<br>Practices around growing season	Crop rotation<br>Potato cultivar selection, fumigation,<br>cover crop, fertilization, pesticide, irrigation

Table 2: Description of variables included in the soil dataset.

Level	# OTUs	# OTUs after filtering
Phylum	57	42
Class	152	108
Order	378	224
Family	476	255
Genus	1319	485

Table 3: Number of OTUs per taxonomic level (first column) in the original data (second column) and after filtering out OTUs that do not appear in at least 15 samples (third column).

Model	Taxonomic levels	Normalization/zero replacement strategies	Number of predictors
ALL-OTU	5	20	42–485
OTU-S0	5	1	7–54
OTU-S1	5	1	5–134
OTU-S2	5	1	5–134
OTU-S3	5	1	7–54
Alpha	5	6	9
Soil	–	6	12
DS	–	6	4

Table 4: Types of predictors per model, number of taxonomic levels, normalization/zero replacement strategies, and range/number of predictors for each type.

	KBest	Mutual	LR	DT	GB	RF	Max	TOTAL
Firmicutes	X	X	X	X	X	X	X	7
Patescibacteria	X	X	X	X	X	X	X	7
Myxococcota	-	-	X	X	X	X	X	5
Methylomirabilota	X	X	X	X	-	-	X	5
Acidobacteriota	-	-	X	X	X	X	X	5
Verrucomicrobiota	-	-	X	X	X	-	X	4
Chloroflexi	-	-	X	X	-	X	X	4
Desulfobacterota	X	X	X	-	-	X	-	4
NB1.j	X	X	-	X	-	-	-	3
Thermoplasmatota	X	X	-	-	X	-	-	3
RCP2.54	-	-	-	X	X	X	-	3
WS2	-	X	-	X	X	-	-	3
Dependentiae	-	-	-	X	X	X	-	3
WPS.2	X	X	-	-	-	X	-	3
Cyanobacteria	-	-	X	-	X	-	X	3
Armatimonadota	X	X	-	X	-	-	-	3
Elusimicrobiota	X	-	-	-	-	X	-	2
Latescibacterota	X	-	-	-	-	X	-	2
Bdellovibrionota	-	-	-	-	X	X	-	2
Bacteroidota	-	-	X	-	-	-	X	2
Spirochaetota	X	X	-	-	-	-	-	2
Nitrospirota	-	-	X	-	-	-	X	2
Actinobacteriota	-	-	X	-	-	-	X	2
Planctomycetota	-	-	-	X	-	X	-	2
Proteobacteria	-	-	X	-	-	-	X	2
SAR324	-	X	-	-	-	-	-	1
Abditibacteriota	-	-	-	-	X	-	-	1
Nitrospinota	-	X	-	-	-	-	-	1
Nanoarchaeota	-	X	-	-	-	-	-	1
Gemmatimonadota	-	-	-	-	-	-	X	1
GAL15	-	-	-	-	X	-	-	1
Deinococcota	X	-	-	-	-	-	-	1
MBNT15	X	-	-	-	-	-	-	1

Table 5: The Operational Taxonomic Units (OTUs) based on their maximum values in samples are sorted and the features that their maximum values are among the top 30% (Max column) are selected. The selected features by the SelectKBest method are marked in the KBest column. Columns three to six are the result of applying of logistic regression (LR), decision tree (DT), Gradient Boosting (GB), or Random Forrest (RF) as the choice of the algorithm using in the Recursive Feature Elimination method. The selected features are also considered based on mutual information statistics as shown in the Mutual column. We assigned a TOTAL value to each OTU based on the number of times the OTU gets picked by any of the seven criteria (six ML models and the max OTU value) shown in the TOTAL column for the Phylum level associated with Scabpit disease. The OTUs with scoring values higher than zero are shown.

	Degree 0	Degree 1	Degree difference
MBNT15	1	9	8
Actinobacteriota	8	3	5
NB1.j	3	8	5
Sva0485	6	2	4
Bacteroidota	4	7	3
Halobacterota	4	1	3
Methylomirabilota	5	2	3
Proteobacteria	3	6	3
Chloroflexi	4	6	2
Cyanobacteria	0	2	2
Entotheonellaeota	3	1	2
GAL15	1	3	2
Hydrogenedentes	2	0	2
Patescibacteria	0	2	2
Planctomycetota	0	2	2
RCP2.54	2	4	2
WPS.2	2	0	2
Zixibacteria	1	3	2
Abditibacteriota	0	1	1
Desulfobacterota	4	5	1
Elusimicrobiota	0	1	1
Fibrobacterota	2	3	1
Gemmatimonadota	2	1	1
Myxococcota	3	2	1
Nanoarchaeota	2	1	1
Nitrospirota	3	2	1
Spirochaetota	3	4	1
Sumerlaeota	1	0	1
Thermoplasmatota	1	0	1
Verrucomicrobiota	0	1	1
Acidobacteriota	3	3	0
Armatimonadota	0	0	0
Bdellovibrionota	2	2	0
Crenarchaeota	1	1	0
Deinococcota	0	0	0
Dependentiae	0	0	0
FCPU426	0	0	0
Firmicutes	1	1	0
Latescibacterota	2	2	0
Nitrospinota	4	4	0
SAR324	0	0	0
WS2	1	1	0

Table 6: List of OTUs in microbial networks constructed by `SPRING` for two classes of scabpit. Degree 0 correspond to the degree of the OTU node in the network built with the samples with Label 0. Degree 1 corresponds to the degree of the OTU node in the network built with samples with Label 1

Parameters	Description	Tuning Parameters
n_estimators	The number of trees in the forest.	[100,200,500]
min_samples_split	Minimum necessary number of samples to split an internal node	[8,10]
min_samples_leaf	The minimum needed the number of samples at a leaf node.	[3,4,5]
max_depth	The maximum depth of the tree	[80,90]
criterion	The function used to evaluate a split's quality	('gini','entropy')

Table 7: Different values for parameters for Random Forest Model.

Description	File name	Time Duration (hour:min:sec)
RF based on OTUs predictors for 20 normalized data and 5 levels	1-RF.py	4:53:02
Feature selection for OTU predictors	2-feature-selectionML.py	1:28:53
RF based on selected OTU and Env features	3-RF-selectedOTU+Env.py	3:07:46
RF based on Environmental features	4-RF-Env.py	5:56:50
RF based on augmented OTUs for 20 normalized data and 5 levels	5-RF-Aug.py	05:09.6
Compare to random based on four strategies	6-RF-compareTorandom.py	16:41.6

Table 8: Running Time for Random Forest.

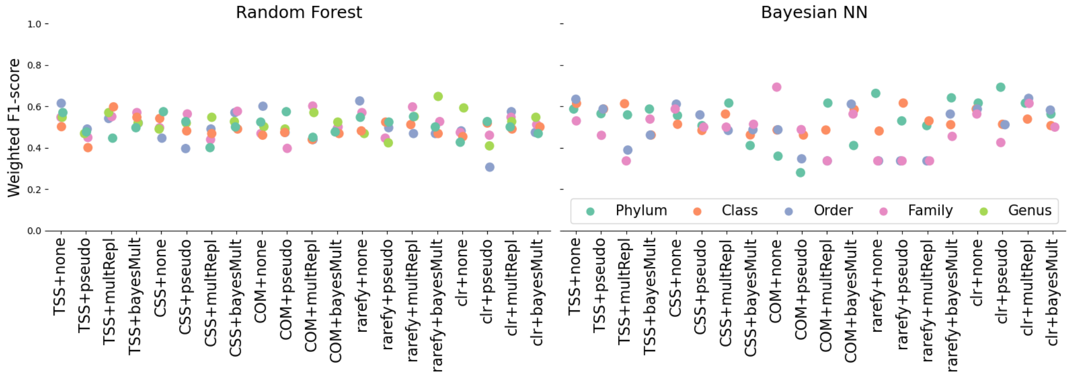

Figure 9: Weighted F1 scores (y-axis) for Random Forest and Bayesian Neural Network (NN) models for yield by meter under the 20 normalization/zero replacement strategies (x-axis). The lack of pattern prevents us from making recommendations of optimal strategies for microbiome OTU data. We can conclude, however, that taxonomic levels, normalization and zero replacement strategies have an effect on the prediction accuracy of the models as evidenced by the broad range displayed by the points.

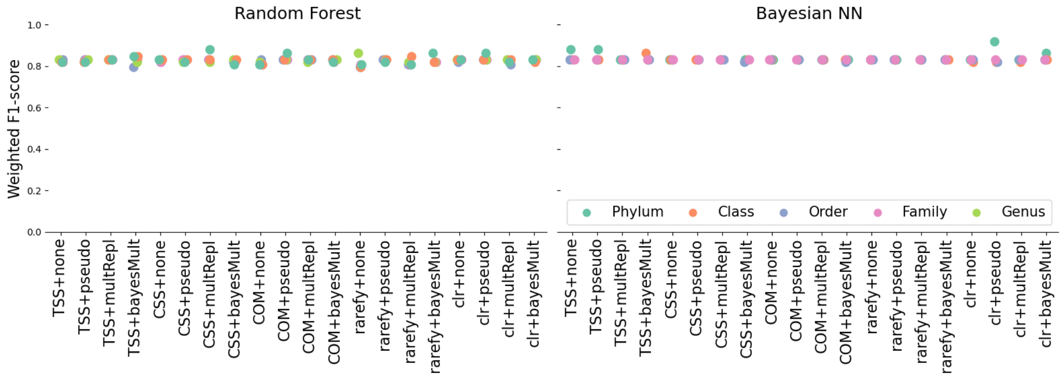

Figure 10: Weighted F1 scores (y-axis) for Random Forest and Bayesian Neural Network (NN) models for black scurf disease under the 20 normalization/zero replacement strategies (x-axis). The lack of pattern prevents us from making recommendations of optimal strategies for microbiome OTU data. We can conclude, however, that taxonomic levels, normalization and zero replacement strategies have an effect on the prediction accuracy of the models as evidenced by the broad range displayed by the points.

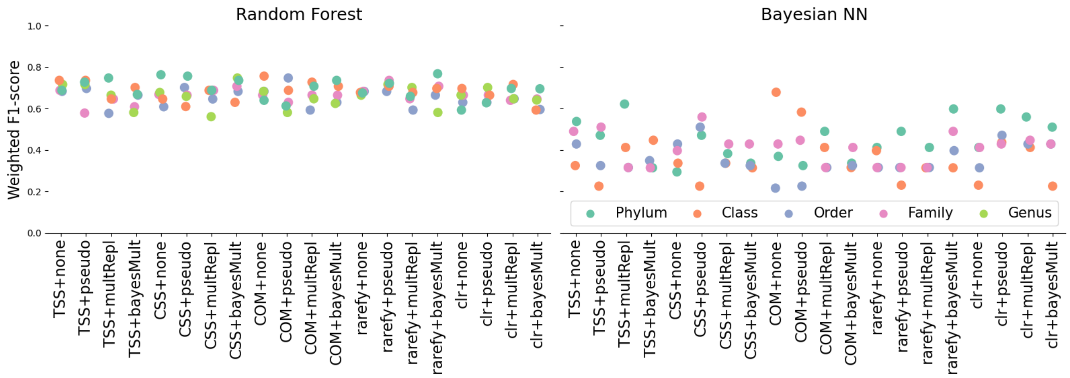

Figure 11: Weighted F1 scores (y-axis) for Random Forest and Bayesian Neural Network (NN) models for scab disease under the 20 normalization/zero replacement strategies (x-axis). The lack of pattern prevents us from making recommendations of optimal strategies for microbiome OTU data. We can conclude, however, that taxonomic levels, normalization and zero replacement strategies have an effect on the prediction accuracy of the models as evidenced by the broad range displayed by the points.

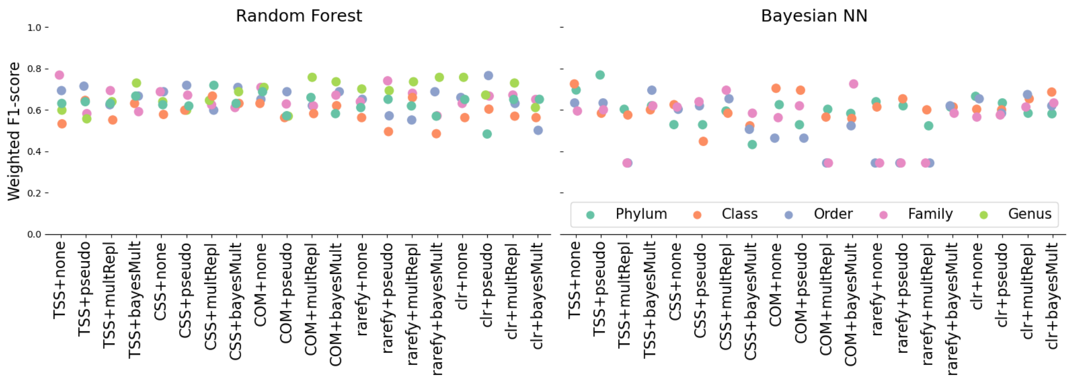

Figure 12: Weighted F1 scores (y-axis) for Random Forest and Bayesian Neural Network (NN) models for superficial scab (Scabsuper) disease under the 20 normalization/zero replacement strategies (x-axis). The lack of pattern prevents us from making recommendations of optimal strategies for microbiome OTU data. We can conclude, however, that taxonomic levels, normalization and zero replacement strategies have an effect on the prediction accuracy of the models as evidenced by the broad range displayed by the points.

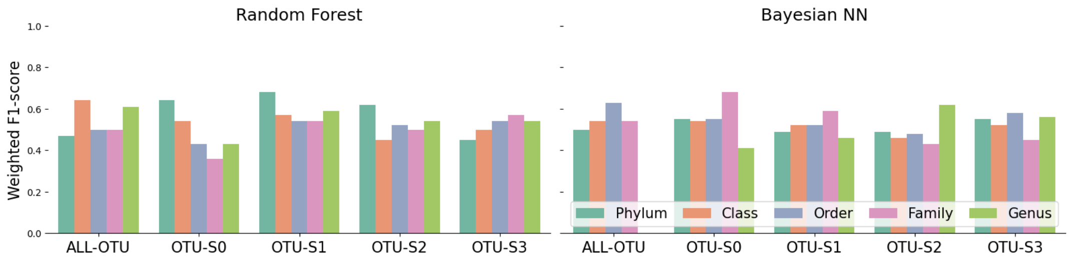

Figure 13: Weighted F1 scores (y-axis) by Random Forest and Bayesian Neural Network (NN) models for yield by plant by feature selection strategy (x-axis) including all OTUs (All-OTU), OTUs selected by the ML method (OTU-S1), the network comparison method (OTU-S2), both methods (OTU-S3), or neither method (OTU-S0).

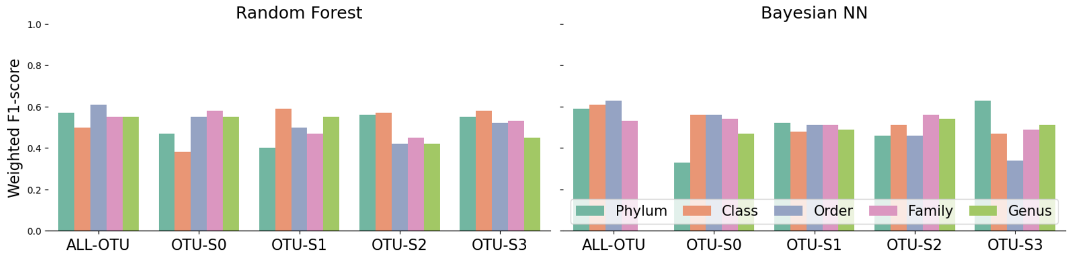

Figure 14: Weighted F1 scores (y-axis) by Random Forest and Bayesian Neural Network (NN) models for yield by meter by feature selection strategy (x-axis) including all OTUs (All-OTU), OTUs selected by the ML method (OTU-S1), the network comparison method (OTU-S2), both methods (OTU-S3), or neither method (OTU-S0).

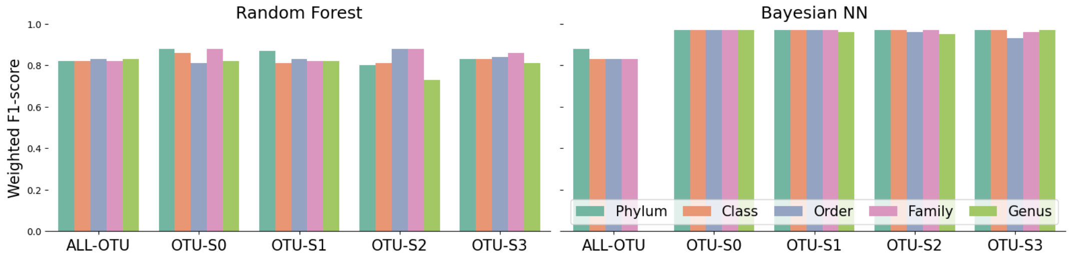

Figure 15: Weighted F1 scores (y-axis) by Random Forest and Bayesian Neural Network (NN) models for black scurf disease by feature selection strategy (x-axis) including all OTUs (All-OTU), OTUs selected by the ML method (OTU-S1), the network comparison method (OTU-S2), both methods (OTU-S3), or neither method (OTU-S0).

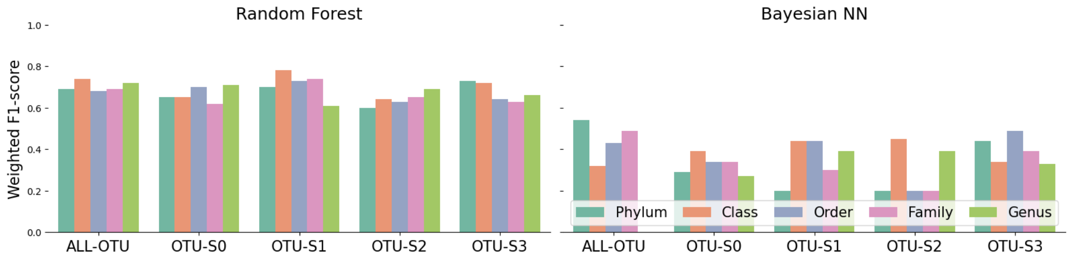

Figure 16: Weighted F1 scores (y-axis) by Random Forest and Bayesian Neural Network (NN) models for scab disease by feature selection strategy (x-axis) including all OTUs (All-OTU), OTUs selected by the ML method (OTU-S1), the network comparison method (OTU-S2), both methods (OTU-S3), or neither method (OTU-S0).

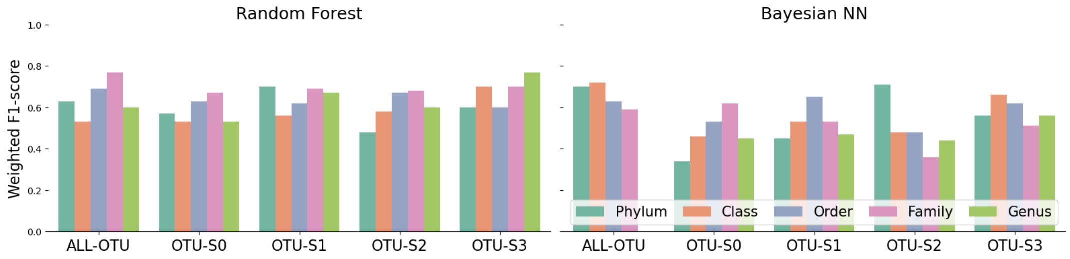

Figure 17: Weighted F1 scores (y-axis) by Random Forest and Bayesian Neural Network (NN) models for superficial scab (Scabsuper) disease by feature selection strategy (x-axis) including all OTUs (All-OTU), OTUs selected by the ML method (OTU-S1), the network comparison method (OTU-S2), both methods (OTU-S3), or neither method (OTU-S0).

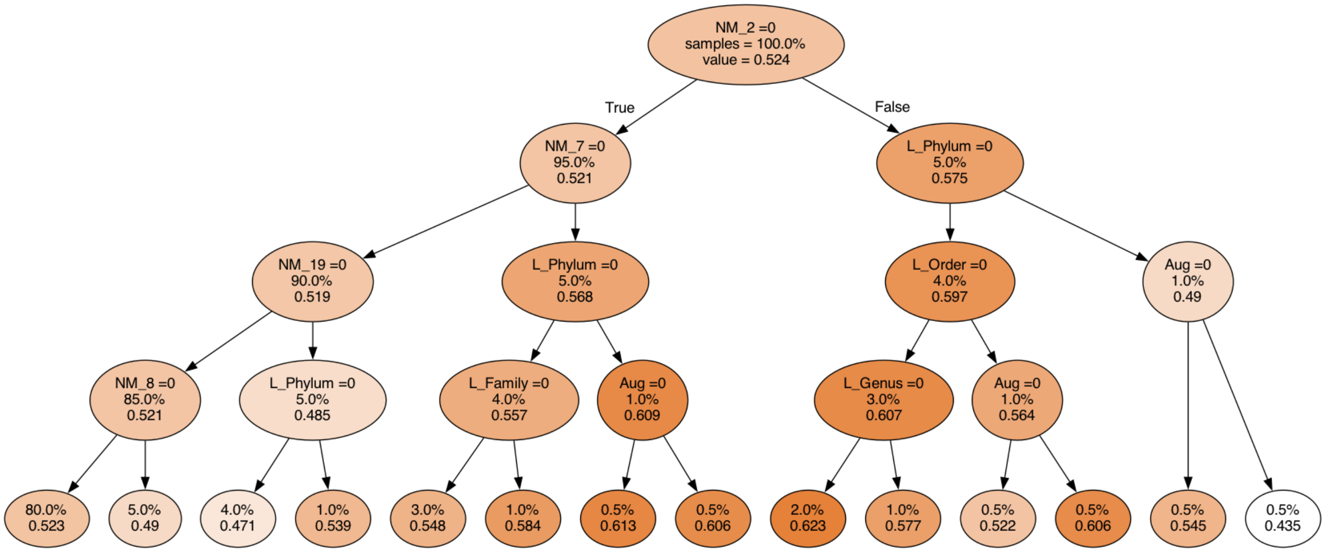

Figure 18: Full model selection decision tree with a maximum depth of 4 summarizing the results of Random Forest models on yield by plant. When the condition at a node is true, we follow the branch on the left, and when the condition is false, we follow the branch on the right. The percentage of data preprocessing options and mean of weighted F1 scores are shown in each nodes.

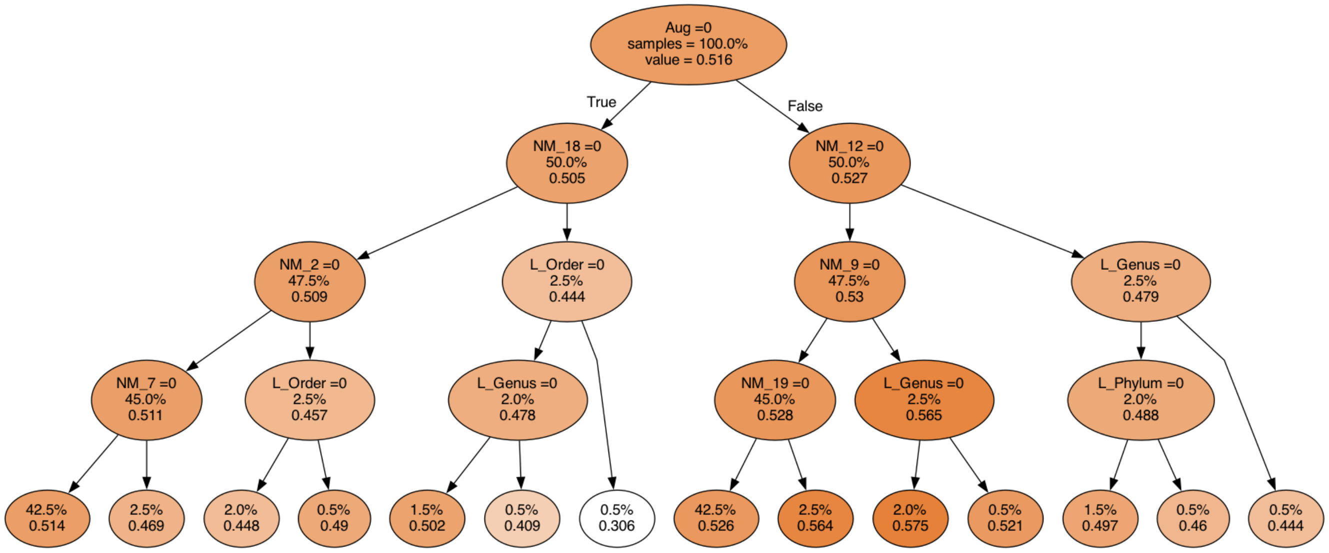

Figure 19: Full model selection decision tree with a maximum depth of 4 summarizing the results of Random Forest models on yield by meter. When the condition at a node is true, we follow the branch on the left, and when the condition is false, we follow the branch on the right. The percentage of data preprocessing options and mean of weighted F1 scores are shown in each nodes.

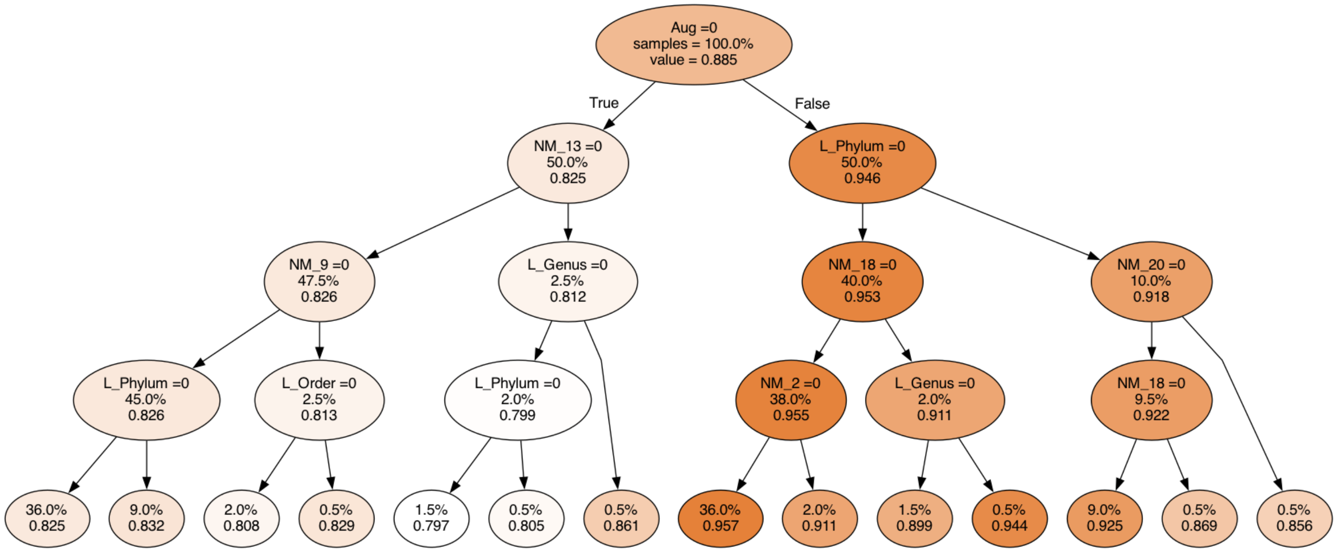

Figure 20: Full model selection decision tree with a maximum depth of 4 summarizing the results of Random Forest models on black scurf disease. When the condition at a node is true, we follow the branch on the left, and when the condition is false, we follow the branch on the right. The percentage of data preprocessing options and mean of weighted F1 scores are shown in each nodes.

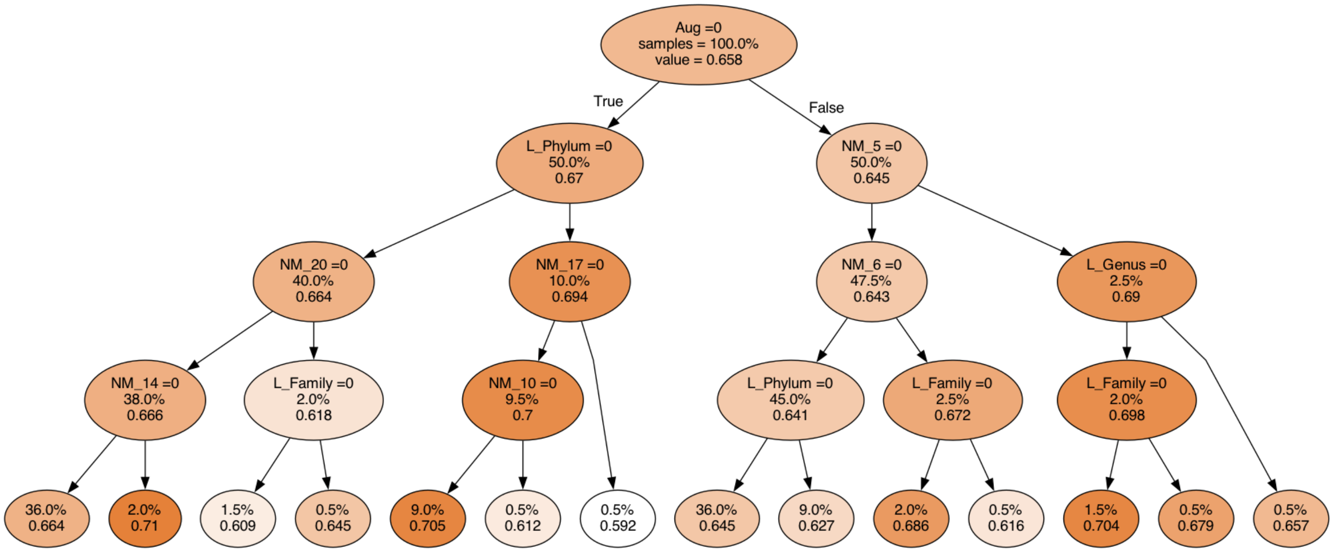

Figure 21: Full model selection decision tree with a maximum depth of 4 summarizing the results of Random Forest models on scab disease. When the condition at a node is true, we follow the branch on the left, and when the condition is false, we follow the branch on the right. The percentage of data preprocessing options and mean of weighted F1 scores are shown in each nodes.

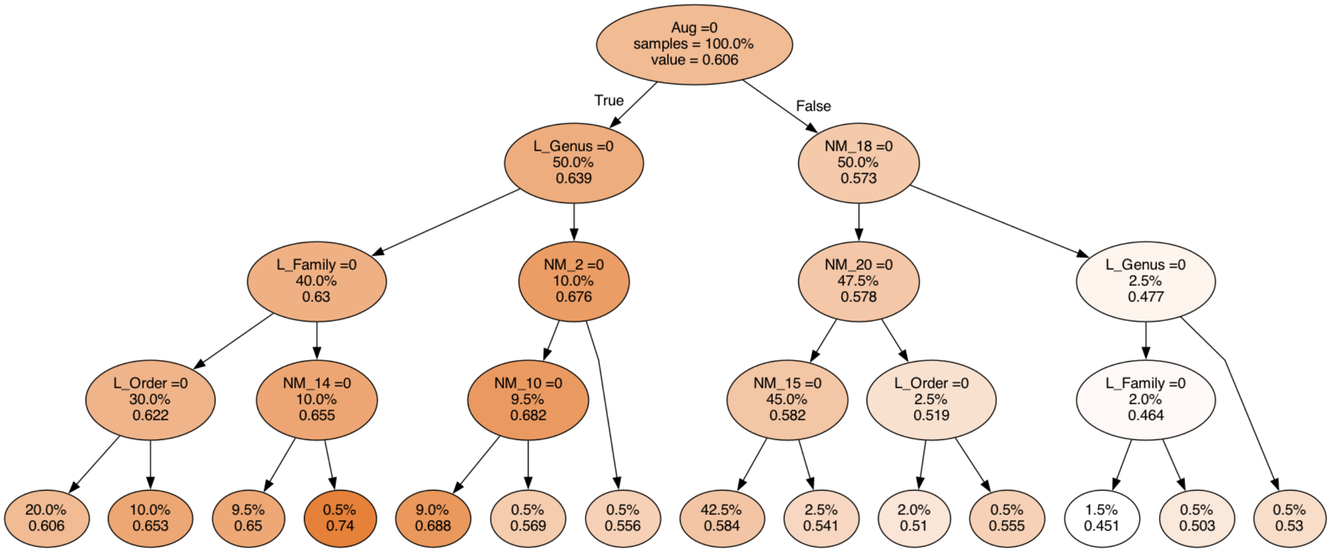

Figure 22: Full model selection decision tree with a maximum depth of 4 summarizing the results of Random Forest models on superficial scab (Scabsuper) disease. When the condition at a node is true, we follow the branch on the left, and when the condition is false, we follow the branch on the right. The percentage of data preprocessing options and mean of weighted F1 scores are shown in each nodes.

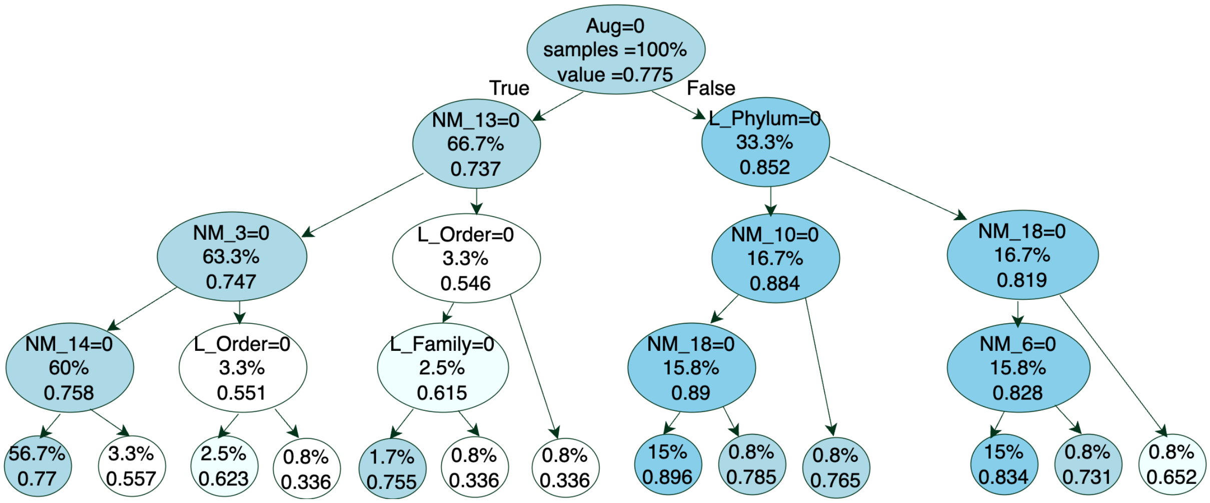

Figure 23: Full model selection decision tree with a maximum depth of 4 summarized the results of Bayesian neural network models on pitted scab disease (Scabpit) disease. When the condition at a node is true, we follow the branch on the left, and when the condition is false, we follow the branch on the right. The percentage of the data preprocessing options and mean of weighted F1 scores are shown in each nodes. The darker the red, the higher the weighted F1 score.

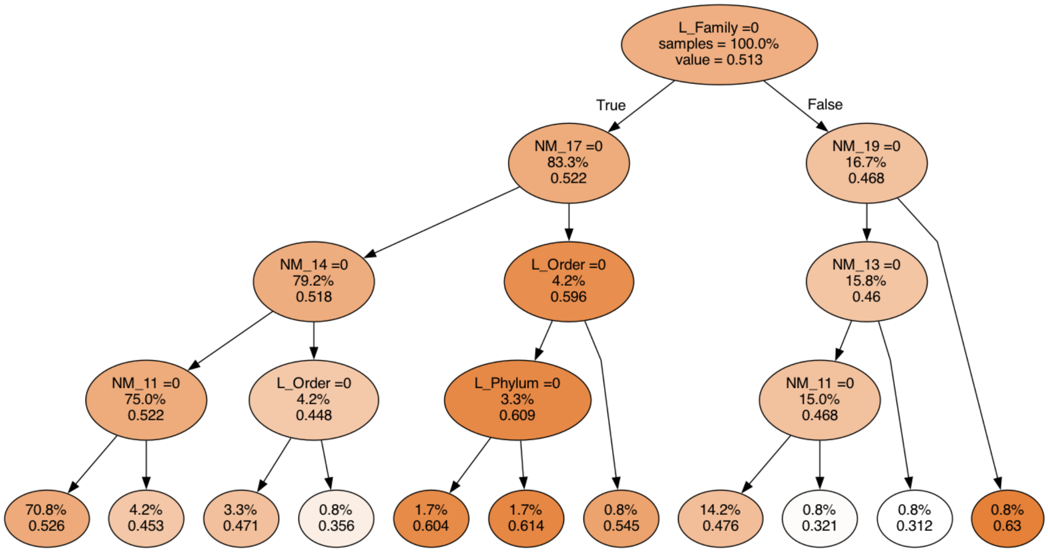

Figure 24: Full model selection decision tree with a maximum depth of 4 summarized the results of Bayesian Neural Network models on yield by plant. When the condition at a node is true, we follow the branch on the left, and when the condition is false, we follow the branch on the right. The percentage of data preprocessing options and mean of weighted F1 scores are shown in each nodes.

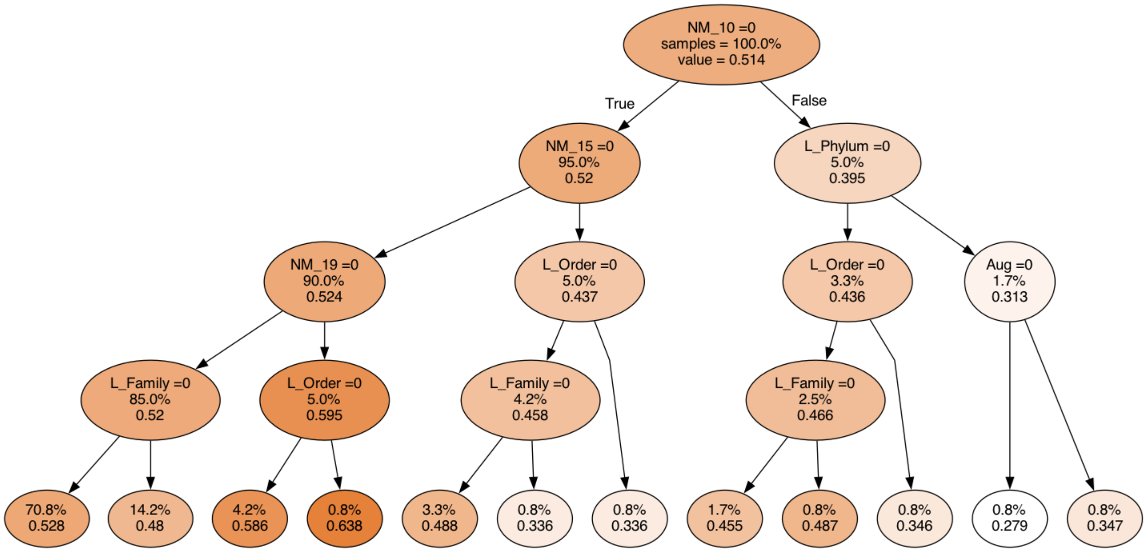

Figure 25: Full model selection decision tree with a maximum depth of 4 summarized the results of Bayesian Neural Network models on yield by meter. When the condition at a node is true, we follow the branch on the left, and when the condition is false, we follow the branch on the right. The percentage of data preprocessing options and mean of weighted F1 scores are shown in each nodes.

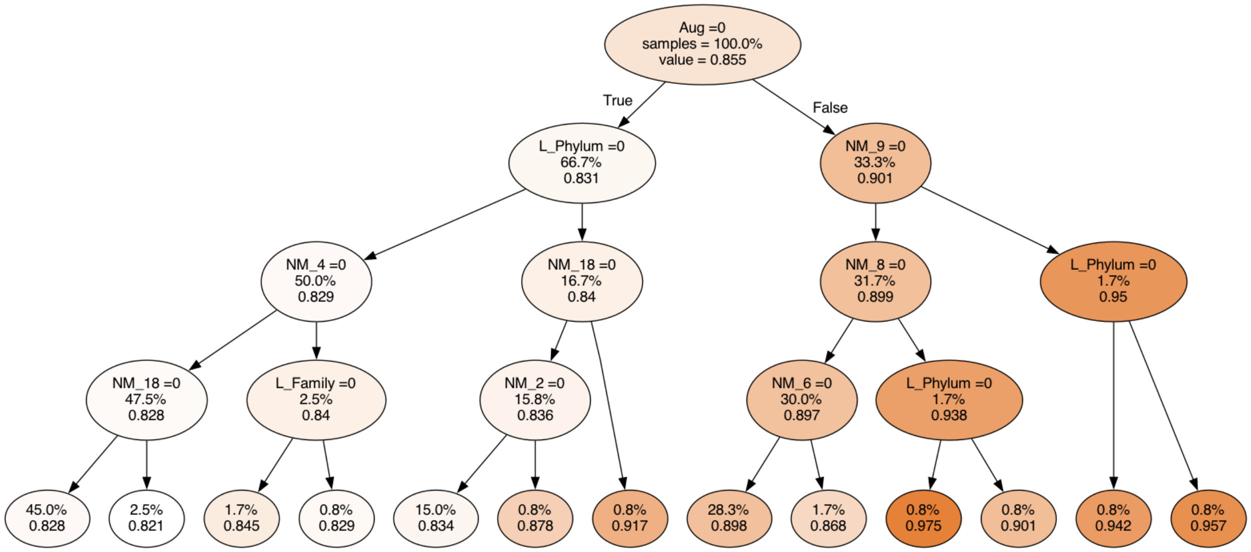

Figure 26: Full model selection decision tree with a maximum depth of 4 summarized the results of Bayesian Neural Network models on black scurf disease. When the condition at a node is true, we follow the branch on the left, and when the condition is false, we follow the branch on the right. The percentage of data preprocessing options and mean of weighted F1 scores are shown in each nodes.

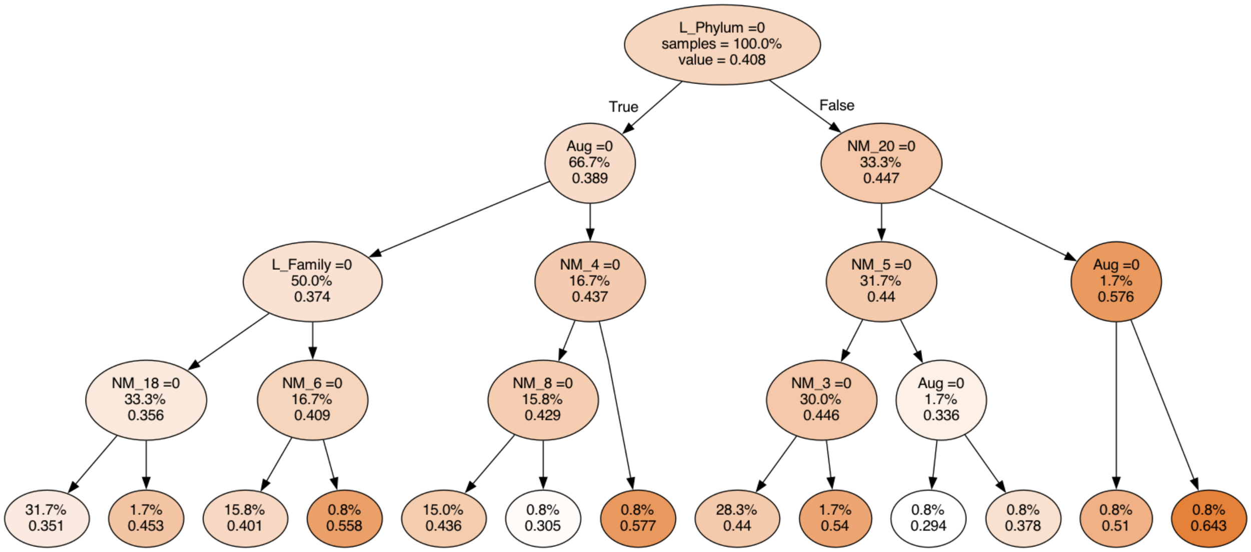

Figure 27: Full model selection decision tree with a maximum depth of 4 summarized the results of Bayesian Neural Network models on scab disease. When the condition at a node is true, we follow the branch on the left, and when the condition is false, we follow the branch on the right. The percentage of data preprocessing options and mean of weighted F1 scores are shown in each nodes.

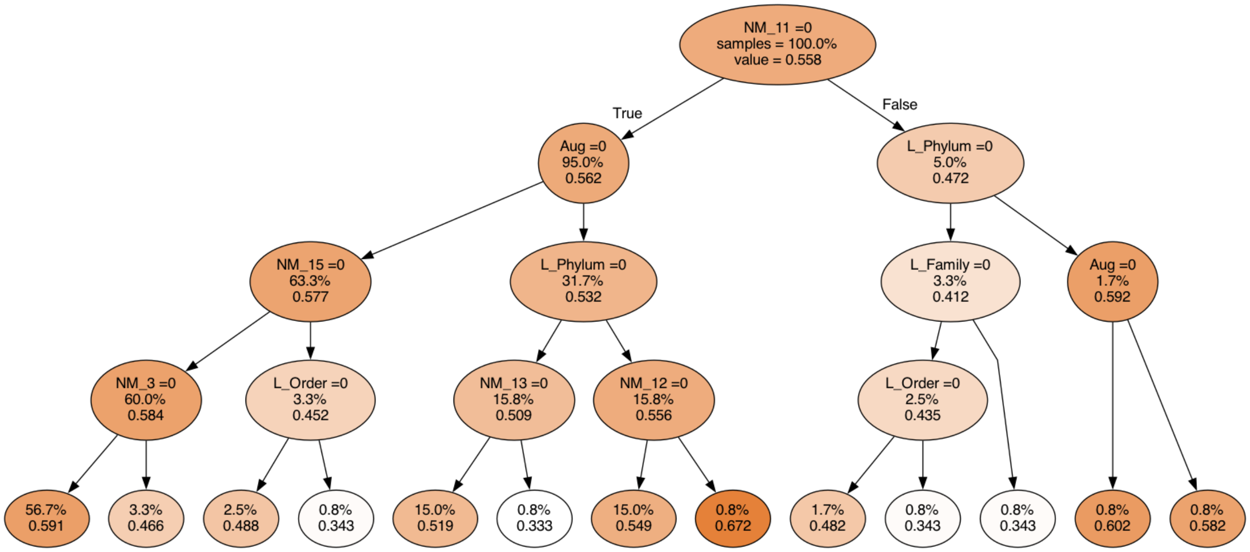

Figure 28: Full model selection decision tree with a maximum depth of 4 summarized the results of Bayesian Neural Network models on superficial scab (Scabsuper) disease. When the condition at a node is true, we follow the branch on the left, and when the condition is false, we follow the branch on the right. The percentage of data preprocessing options and mean of weighted F1 scores are shown in each nodes.

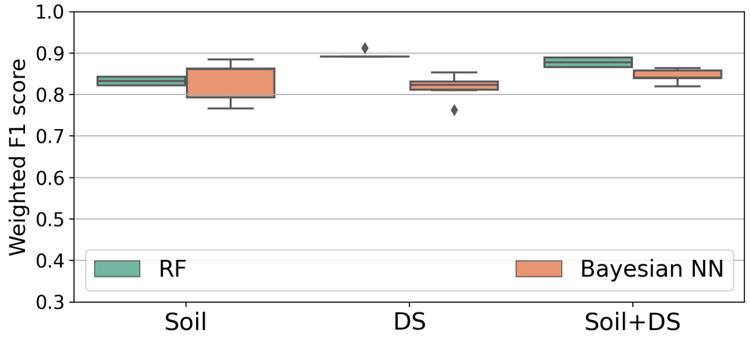

Figure 29: Boxplots of the weighted F1 scores by random forest (RF) and Bayesian neural network (Bayesian NN) models for pitted scab disease using environmental predictors (Soil: Soil chemistry, DS: Microbial population density in soil).

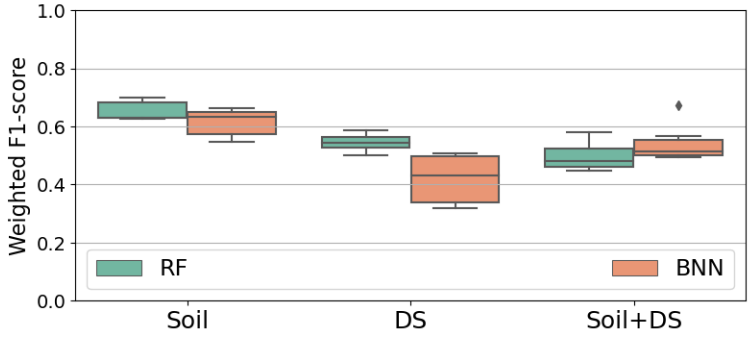

Figure 30: Boxplots of the weighted F1 scores by Random Forest (RF) and Bayesian Neural Network (BNN) models for yield by plant using environmental predictors (Soil: Soil chemistry, DS: Microbial population density in soil).

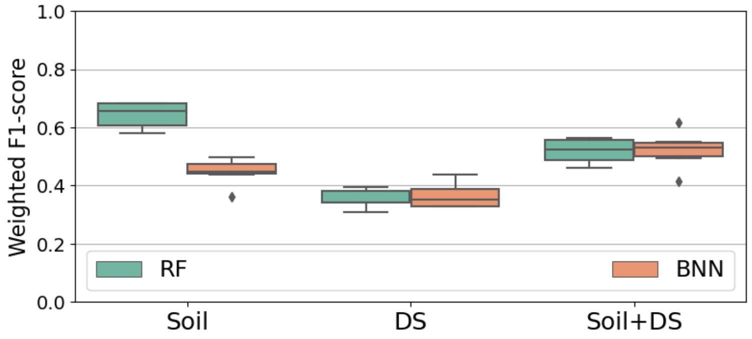

Figure 31: Boxplots of the weighted F1 scores by Random Forest (RF) and Bayesian Neural Network (BNN) models for yield by meter using environmental predictors (Soil: Soil chemistry, DS: Microbial population density in soil).

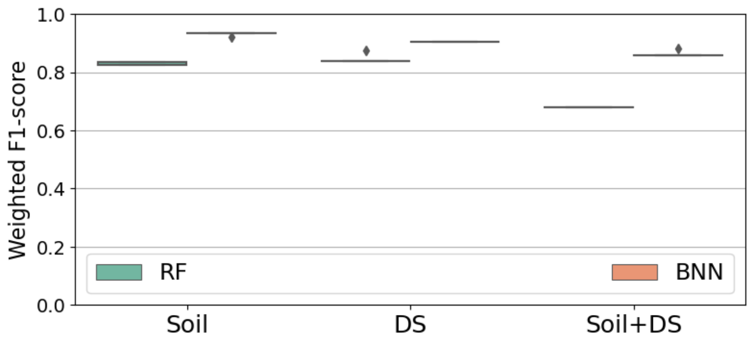

Figure 32: Boxplots of the weighted F1 scores by Random Forest (RF) and Bayesian Neural Network (BNN) models for black scurf disease using environmental predictors (Soil: Soil chemistry, DS: Microbial population density in soil).

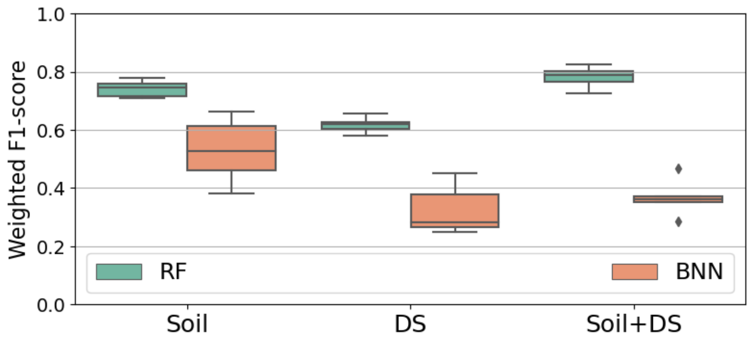

Figure 33: Boxplots of the weighted F1 scores by Random Forest (RF) and Bayesian Neural Network (BNN) models for scab disease outcome using environmental predictors (Soil: Soil chemistry, DS: Microbial population density in soil).

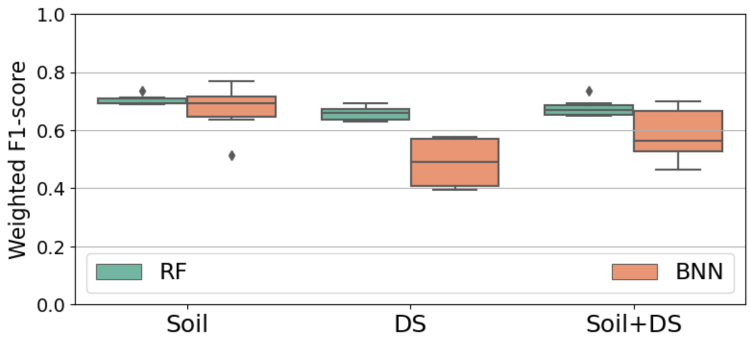

Figure 34: Boxplots of the weighted F1 scores by Random Forest (RF) and Bayesian Neural Network (BNN) models for superficial scab (Scabsuper) disease using environmental predictors (Soil: Soil chemistry, DS: Microbial population density in soil).

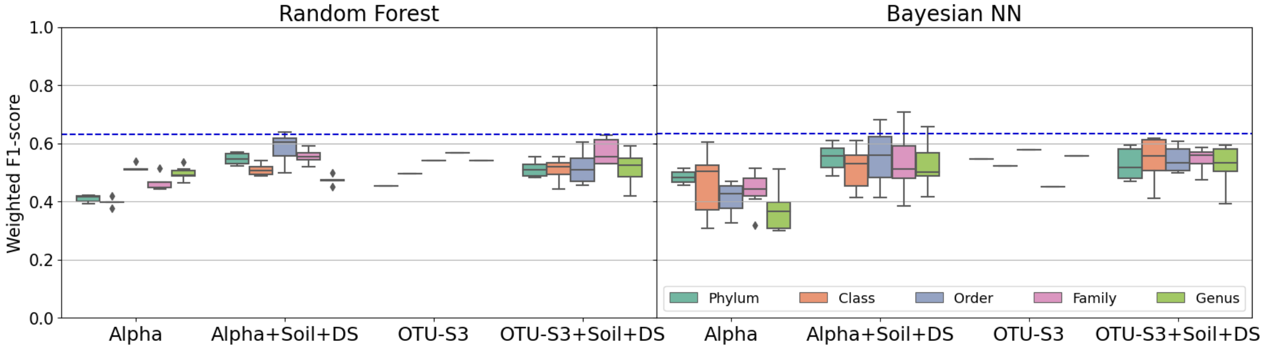

Figure 35: Boxplots with the weighted F1 scores (y-axis) by Random Forest and Bayesian Neural Network (NN) models for yield per plant. The models including both types of predictors outperform other models, yet models including OTU data alone (OTU-S3) are comparable which suggests that the microbial information indeed contains signal to predict the disease outcome on its own. However, OTU data is more expensive to collect, and perhaps not necessary, given that the model without OTU data (Soil) performs just as accurately (blue dashed line).

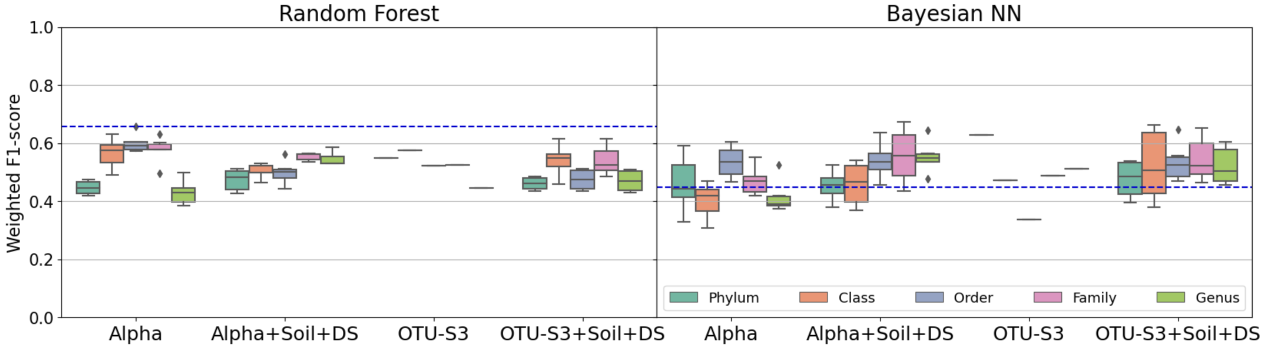

Figure 36: Boxplots with the weighted F1 scores (y-axis) by Random Forest and Bayesian Neural Network (NN) models for yield per meter. The models including both types of predictors outperform other models, yet models including OTU data alone (OTU-S3) are comparable which suggests that the microbial information indeed contains signal to predict the disease outcome on its own. However, OTU data is more expensive to collect, and perhaps not necessary, given that the model without OTU data (Soil) performs just as accurately (blue dashed line).

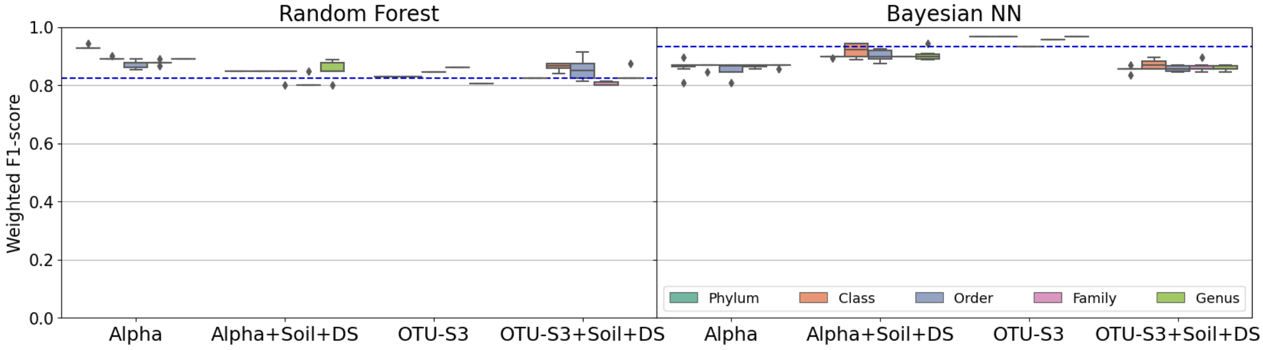

Figure 37: Boxplots with the weighted F1 scores (y-axis) by Random Forest and Bayesian Neural Network (NN) models for black scurf disease. The models including both types of predictors outperform other models, yet models including OTU data alone (OTU-S3) are comparable which suggests that the microbial information indeed contains signal to predict the disease outcome on its own. However, OTU data is more expensive to collect, and perhaps not necessary, given that the model without OTU data (Soil) performs just as accurately (blue dashed line).

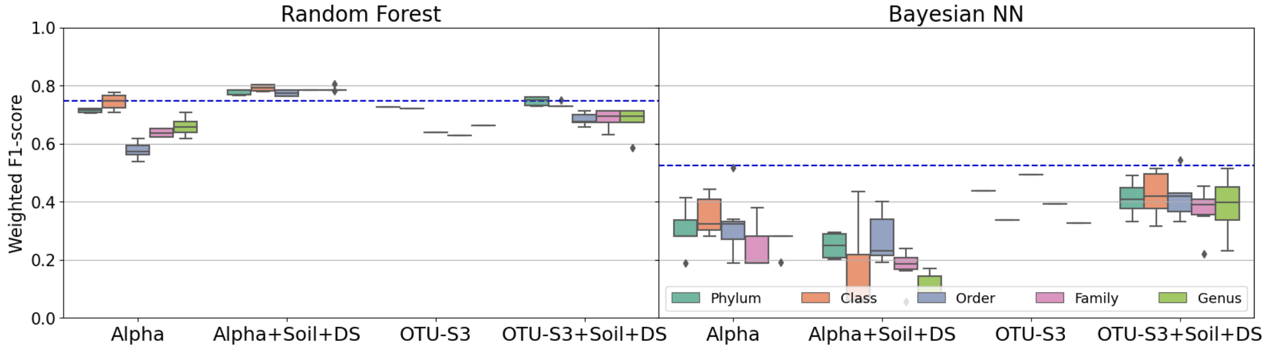

Figure 38: Boxplots with the weighted F1 scores (y-axis) by Random Forest and Bayesian Neural Network (NN) models for scab disease. The models including both types of predictors outperform other models, yet models including OTU data alone (OTU-S3) are comparable which suggests that the microbial information indeed contains signal to predict the disease outcome on its own. However, OTU data is more expensive to collect, and perhaps not necessary, given that the model without OTU data (Soil) performs just as accurately (blue dashed line).

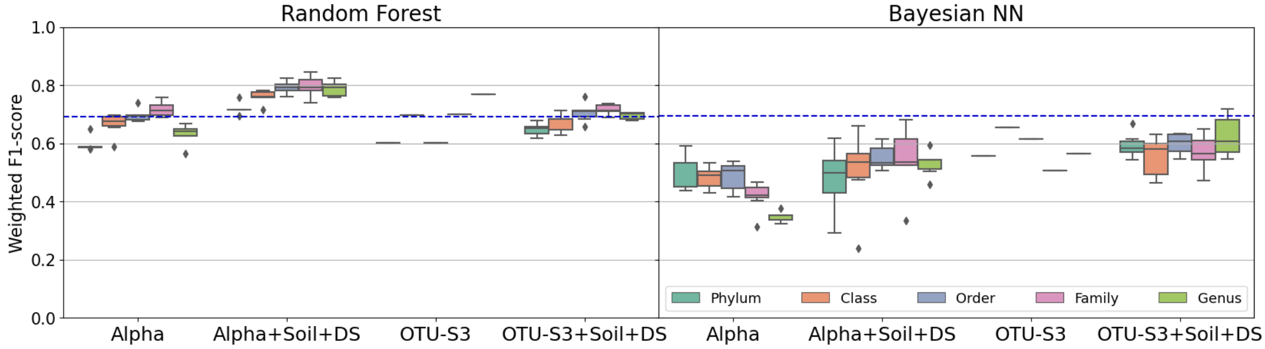

Figure 39: Boxplots with the weighted F1 scores (y-axis) by Random Forest and Bayesian Neural Network (NN) models for superficial scab (Scabsuper) disease. The models including both types of predictors outperform other models, yet models including OTU data alone (OTU-S3) are comparable which suggests that the microbial information indeed contains signal to predict the disease outcome on its own. However, OTU data is more expensive to collect, and perhaps not necessary, given that the model without OTU data (Soil) performs just as accurately (blue dashed line).
